# Binuclear Triphenylantimony(V) Catecholates through N-Donor Linkers: Structural Features and Redox Properties

**DOI:** 10.3390/molecules27196484

**Published:** 2022-10-01

**Authors:** Andrey I. Poddel’sky, Ivan V. Smolyaninov, Aleksandra I. Shataeva, Evgenii V. Baranov, Georgy K. Fukin

**Affiliations:** 1 G.A. Razuvaev Institute of Organometallic Chemistry, Russian Academy of Sciences, 49 Tropinina Str., 603137 Nizhny Novgorod, Russia; 2 Department of Chemistry, Astrakhan State Technical University, 16 Tatisheva Str., 414056 Astrakhan, Russia

**Keywords:** antimony(V), catecholate, binuclear complex, N-donor linker ligand, single-crystal X-ray, cyclic voltammetry

## Abstract

A series of binuclear triphenylantimony(V) bis-catecholato complexes **1**–**11** of the type (Cat)Ph_3_Sb-linker-SbPh_3_(Cat) was prepared by a reaction of the corresponding mononuclear catecholates (Cat)SbPh_3_ with a neutral bidentate donor linker ligands pyrazine (Pyr), 4,4′-dipyridyl (Bipy), bis-(pyridine-4-yl)-disulfide (PySSPy), and diazobicyclo[2,2,2]octane (DABCO) in a dry toluene: Cat = 3,6-di-tert-butyl-catecholate (3,6-DBCat), linker = Pyr (**1**); PySSPy (**2**); Bipy (**3**); DABCO (**4**); Cat = 3,5-di-tert-butyl-catecholate (3,5-DBCat), linker = Bipy (**5**); DABCO (**9**); Cat = 4,5-(piperazine-1,4-diyl)-3,6-di-tert-butylcatecholate (pip-3,6-DBCat), linker = Bipy (**6**); DABCO (**10**); Cat = 4,5-dichloro-3,6-di-tert-butylcatecholate (4,5-Cl_2_-3,6-DBCat), linker = Bipy (**7**); DABCO (**11**); and Cat = 4,5-dimethoxy-3,6-di-tert-butylcatecholate (4,5-(MeO)_2_-3,6-DBCat), linker = Bipy (**8**). The same reaction of (4,5-Cl_2_-3,6-DBCat)SbPh_3_ with DABCO in an open atmosphere results in a formation of 1D coordination polymer {[(4,5-Cl_2_-3,6-DBCat)SbPh_3_·H_2_O]·DABCO}_n_ (**12**). Bis-catecholate complex Ph_3_Sb(Cat-Spiro-Cat)SbPh_3_ reacts with Bipy as 1:1 yielding a rare macrocyclic tetranuclear compound {Ph_3_Sb(Cat-Spiro-Cat)SbPh_3_∙(Bipy)}_2_ (**13**). The molecular structures of **1**, **3**, **4**, **5**, **8**, **10**, **12**, and **13** in crystal state were established by single-crystal X-ray analysis. Complexes demonstrate different types of relative spatial positions of mononuclear moieties. The nature of chemical bonds, charges distribution, and the energy of Sb...N interaction were investigated in the example of complex **5**. The electrochemical behavior of the complexes depends on the coordinated N-donor ligand. The coordination of pyrazine, Bipy, and PySSPy at the antimony atom changes their mechanism of electrooxidation: instead of two successive redox stages Cat/SQ and SQ/Cat, one multielectron stage was observed. The coordination of the DABCO ligand is accompanied by a significant shift in the oxidation potentials of the catecholate ligand to the cathodic region (by 0.4 V), compared to the initial complex.

## 1. Introduction

Organoantimonials represent a very diverse class of antimony compounds, attracting growing attention from scientists because of the variety of structural motifs offered by this type compounds (including acting as ligands in different complexes) [1,2,3,4,5,6,7,8,9,10,11], various and sometimes unusual reactivity [12,13,14,15,16,17,18,19,20,21], and prospective applications in biochemistry and medicine [20,21,22,23,24,25,26,27,28,29,30]. So, some tetraarylstibonium cations, as well as cationic stibine–palladium complexes, may be used as sensors for the toxic fluoride anion, even in water [31,32,33,34], can activate the Element–Fluorine bond and promote the hydrodefluorination of fluoroalkanes in the presence of Et_3_SiH [35], or activate the Ag–Cl bond in gold chloride complex to catalyze the cyclization of propargyl amides [36]. A series of tetraarylstibonium cations have been evaluated as catalysts for the cycloaddition of oxiranes and isocyanates under mild conditions [37], the selective catalytic transformation of aldehydes into different products (symmetric ethers, α,β-unsaturated aldehydes, 1,3,5-trioxanes), depending on conditions [38,39]. Some antimony–platinum complexes reveal the coordination non-innocence of antimony ligands, which can be exploited for the purpose of electrophilic catalysis in enyne cyclization or intramolecular hydroarylation reactions [40,41,42].

Triarylantimony(V) catecholato derivatives Sb(C_6_F_5_)_3_(O_2_C_6_Cl_4_), as well as triptycene-based distiborane bis-catecholate possess strong Lewis acidic properties [43,44,45,46]. Triarylantimony(V) o-amidophenolates [47,48], as well as some triarylantimony(V) catecholates with electron-donor substituents [49,50,51], represent the first class of main group metal complexes, which can bind the molecular oxygen in a reversible manner. The related antimony(V) catecholates were found to be active in a radical scavenging [52,53,54], can be considered prospective inhibitors of oxidation processes, such as lipid peroxidation [55,56]. The modification of molecular structure and redox-properties of triorganylantimony(V) catecholates is possible via the use of different donor/acceptor substituents in a redox-active ligand [50,57], at organic groups bound to antimony [58,59,60], or coordination of a neutral or ionic ligand to the central antimony atom [61,62,63].

Implementing various types of coordination spheres of antimony(V) complexes and the realization of complex supramolecular structures with various types of binding allows us to consider antimony(V) catecholates as model objects for the construction of bi- or polynuclear compounds using bidentate type ligands. In the present paper, we have applied such bidentate linker ligands as pyrazine, 4,4′-dipyridyl, bis-(pyridine-4-yl)-disulfide, and diazobicyclo[2,2,2]octane to construct antimony(V) catecholato complexes of the type (Cat)Ph_3_Sb-linker-SbPh_3_(Cat).

## 2. Results and Discussion

### 2.1. Synthesis and Characterization

**Scheme 1:**
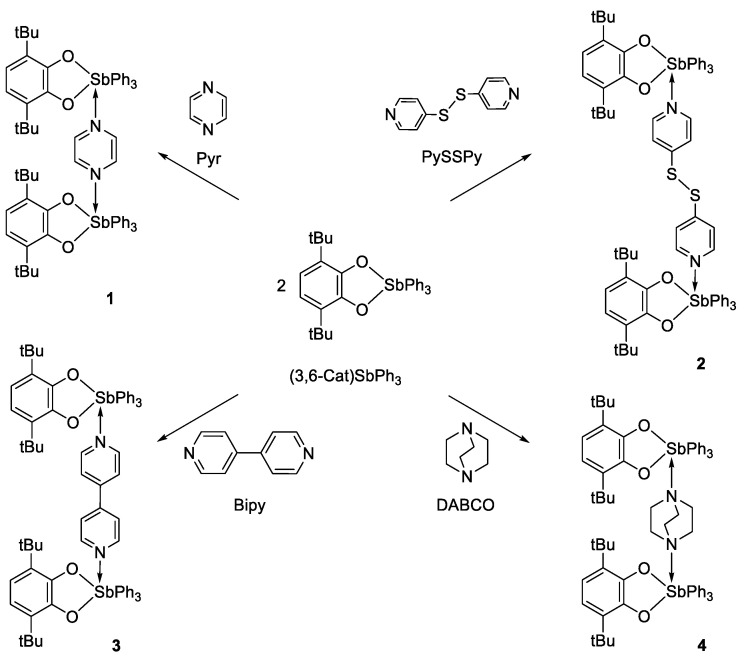
Preparation of binuclear triphenylantimony(V) catecholates **1**–**4**.

In general, binuclear triphenylantimony(V) bis-catecholates of the type (Cat)SbPh_3_-linker-Ph_3_Sb(Cat), where linker is a bidentate nitrogen donor ligand, can be easily prepared: the treatment of (3,6-DBCat)SbPh_3_ with pyrazine (Pyr), bis-(pyridine-4-yl)-disulfide (PySSPy), 4,4′-bipyridyl (Bipy), and diazobicyclo[2,2,2]octane (DABCO) in a non-coordinating solvent leads to the formation of the corresponding binuclear complexes **1**–**4**, where these nitrogen-containing bases play a role of linker ligand (Scheme 1).

**Scheme 2:**
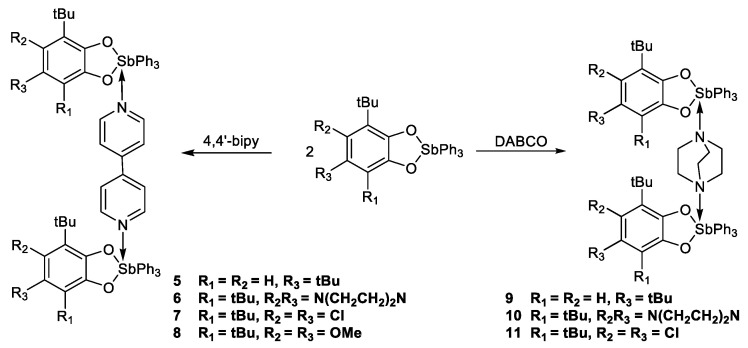
Preparation of binuclear triphenylantimony(V) catecholates **5**–**11**.

The use of other substituted triphenylantimony(V) catecholates: 3,5-di-tert-butylcatecholate, 4,5-(piperazine-1,4-diyl)-3,6-di-tert-butylcatecholate, 4,5-dichloro-3,6-di-tert-butylcatecholate, 4,5-dimethoxy-3,6-di-tert-butylcatecholate, i.e., catecholates with different substituents at the 4th–6th positions of aromatic catecholate ring, does not prevent the formation of binuclear complexes. A row of bis-catecholates **5**–**11** of triphenylantimony(V) with Bipy and DABCO were also synthesized by the simple addition of ligand to the corresponding mononuclear triphenylantimony(V) catecholate in a warm toluene in the absence of moisture (Scheme 2).

In all cases, subsequent to the experimental manipulations, a slow evaporation of the solvent (toluene, n-hexane, or a mixture of toluene-hexane) allowed the obtaining of light yellow to orange crystalline powders of complexes **1**–**11**.

**Scheme 3:**
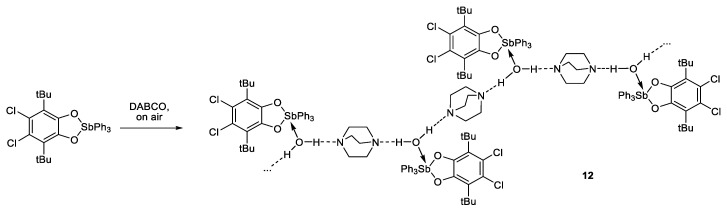
Reaction of (4,5-Cl_2_-3,6-DBCat)SbPh_3_ with DABCO in an open atmosphere.

The syntheses shown in Scheme 1 and Scheme 2 should be performed in a dry atmosphere in order to exclude the coordination of water to antimony atom with a formation of complexes of the other types, as it was shown on the example of reaction between triphenylantimony(V) 4,5-dichloro-3,6-di-tert-butylcatecholate with DABCO in an open atmosphere (Scheme 3). The crystallization of this reaction mixture in an open atmosphere leads to the formation of a coordination polymeric complex of the type {[(4,5-Cl_2_-3,6-DBCat)SbPh_3_·H_2_O]·DABCO}_n_ (**12**).

**Scheme 4:**
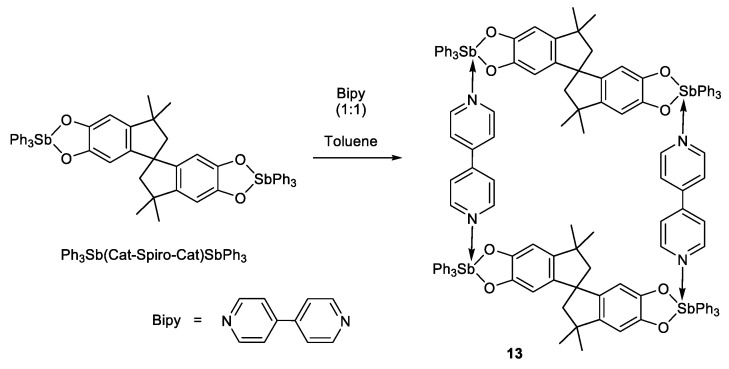
Synthesis of tetranuclear complex {Ph_3_Sb(Cat-Spiro-Cat)SbPh_3_∙(Bipy)}_2_ (**13**).

Another interesting type—tetranuclear antimony(V) catecholate—may be prepared starting from Bipy and triphenylantimony(V) spiro-bis-catecholate Ph_3_Sb(Cat-Spiro-Cat)SbPh_3_ in an equimolar ratio (Scheme 4). Complex {Ph_3_Sb(Cat-Spiro-Cat)SbPh_3_∙(Bipy)}_2_ (**13**) was isolated as light orange crystals **13**∙6toluene suitable for single-crystal X-ray analysis.

The structure of **1**–**13** was confirmed by the IR spectroscopy, ^1^H, ^13^C{^1^H} NMR spectroscopy (Supplementary Materials (ESI): Figures S1–S26), and elemental analysis. The formation of complexes in solution is confirmed by ^1^H and ^13^C{^1^H} NMR spectroscopy. For example, the signals from protons of linker ligand in the ^1^H NMR spectra of complexes **1** and **2** in CDCl_3_ solution undergo a shift in comparison with the signals of free ligands: singlet at δ = 8.54 ppm for **1** and δ = 8.59 ppm for uncoordinated pyrazine; multiplets at δ = 8.45 and 7.30 ppm for **2**; and at δ = 8.55 and 7.25 ppm for free bis-(pyridine-4-yl)-disulfide, respectively.

According to IR spectroscopy, the presence of the coordinated neutral N-donor ligands in the complexes is determined by the stretching vibrations *v*(C_arom_-N) on the region of 1360–1250 cm^−1^ and stretching vibrations *v*(C-N) in the range of 1200–1240 cm^−1^.

### 2.2. X-ray Structures

**Figure 1:**
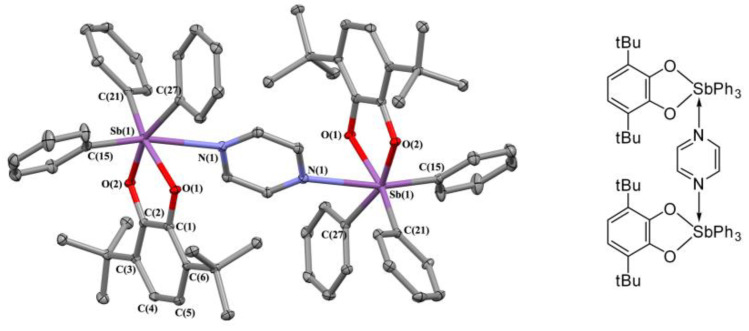
The X-ray structure of **1** (thermal ellipsoids here and below of 50% probability). The hydrogen atoms are omitted. The selected bond lengths (Ǻ) and angles (°): Sb(1)-O(1) 2.0194(12), Sb(1)-O(2) 2.0326(12), Sb(1)-N(1) 2.773(5), Sb(1)-C(15) 2.1246(19), Sb(1)-C(21) 2.1433(18), Sb(1)-C(27) 2.1434(18), O(1)-C(1) 1.353(2), O(2)-C(2) 1.361(2); O(1)-Sb(1)-O(2) 79.69(5), O(1)-Sb(1)-C(21) 155.31(6), O(2)-Sb(1)-C(27) 160.75(6), N(1)-Sb(1)-C(15) 167.87(6).

**Figure 2:**
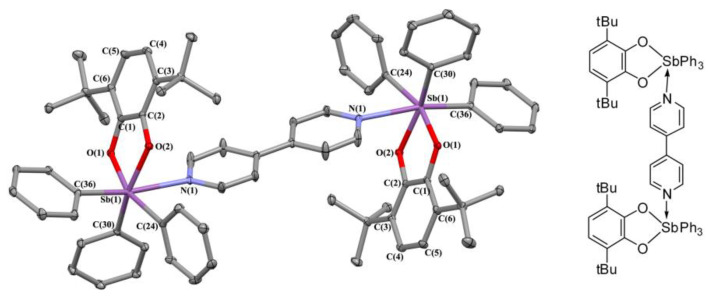
The X-ray structure of **3**. The hydrogen atoms are omitted. The selected bond lengths (Ǻ) and angles (°): Sb(1)-O(1) 2.0388(7), Sb(1)-O(2) 2.0359(7), Sb(1)-N(1) 2.5808(8), Sb(1)-C(24) 2.1429(10), Sb(1)-C(30) 2.1505(10), Sb(1)-C(36) 2.1283(10), O(1)-C(1) 1.3628(12), O(2)-C(2) 1.3607(12); O(2)-Sb(1)-O(1) 79.55(3), O(1)-Sb(1)-C(24) 161.23(3), O(2)-Sb(1)-C(30) 157.70(3), N(1)-Sb(1)-C(36) 170.87(3).

**Figure 3:**
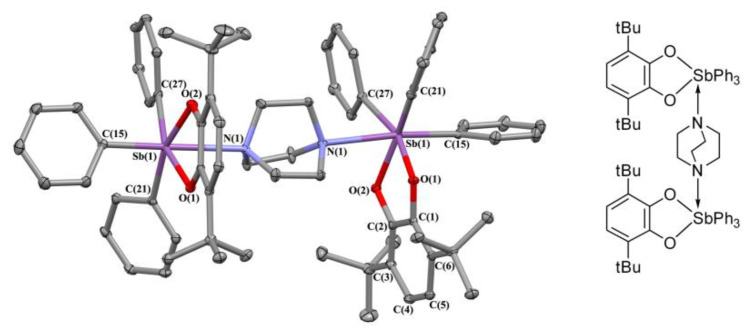
The X-ray structure of **4**. The hydrogen atoms and methyl groups of tert-butyls are omitted. The selected bond lengths (Ǻ) and angles (°): Sb(1)-O(1) 2.0360(10), Sb(1)-O(2) 2.0405(10), Sb(1)-N(1) 2.645(9), Sb(1)-C(15) 2.1413(14), Sb(1)-C(21) 2.1475(14), Sb(1)-C(27) 2.1547(15), O(1)-C(1) 1.3672(17), O(2)-C(2) 1.3618(17), O(1)-Sb(1)-O(2) 79.26(4), O(2)-Sb(1)-C(21) 163.97(5), O(1)-Sb(1)-C(27) 161.80(5), N(1)-Sb(1)-C(15) 175.09(5).

**Figure 4:**
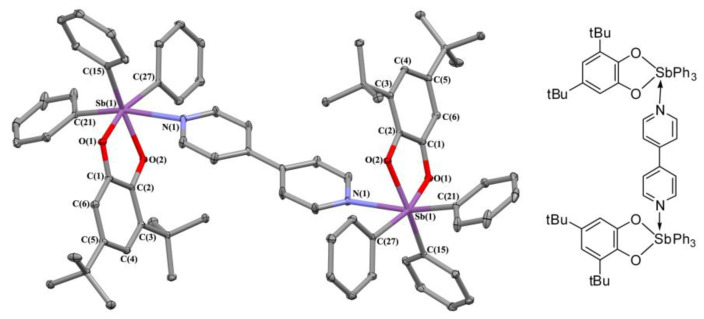
The X-ray structure of **5**. The hydrogen atoms are omitted. The selected bond lengths (Ǻ) and angles (°): Sb(1)-O(1) 2.041(2), Sb(1)-O(2) 2.039(2), Sb(1)-N(1) 2.604(3), Sb(1)-C(15) 2.161(3), Sb(1)-C(21) 2.135(3), Sb(1)-C(27) 2.142(3), O(1)-C(1) 1.360(4), O(2)-C(2) 1.364(4), O(1)-Sb(1)-O(2) 80.36(9), O(1)-Sb(1)-C(27) 163.89(10), O(2)-Sb(1)-C(15) 157.98(10), N(1)-Sb(1)-C(21) 167.78(11).

**Figure 5:**
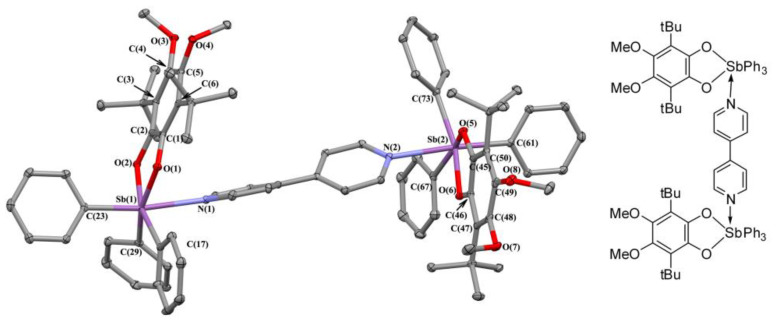
The X-ray structure of **8**. The hydrogen atoms are omitted. The selected bond lengths (Ǻ) and angles (°): Sb(1)-O(1) 2.0316(17), Sb(1)-O(2) 2.0291(17), Sb(2)-O(5) 2.0429(17), Sb(2)-O(6) 2.0415(16), Sb(1)-N(1) 2.494(2), Sb(2)-N(2) 2.520(2), Sb(1)-C(17) 2.151(2), Sb(1)-C(23) 2.129(2), Sb(1)-C(29) 2.146(2), Sb(2)-C(61) 2.132(2), Sb(2)-C(67) 2.154(3), Sb(2)-C(73) 2.141(2), O(1)-C(1) 1.368(3), O(2)-C(2) 1.364(3), O(5)-C(45) 1.357(3), O(6)-C(46) 1.363(3), O(1)-Sb(1)-O(2) 78.78(7), O(1)-Sb(1)-C(29) 159.20(8), O(2)-Sb(1)-C(17) 163.51(8), N(1)-Sb(1)-C(23) 171.36(8), O(5)-Sb(2)-O(6) 78.19(7), O(5)-Sb(2)-C(67) 159.63(8), O(6)-Sb(2)-C(73) 156.80(8), N(2)-Sb(2)-C(61) 175.53(8).

**Figure 6:**
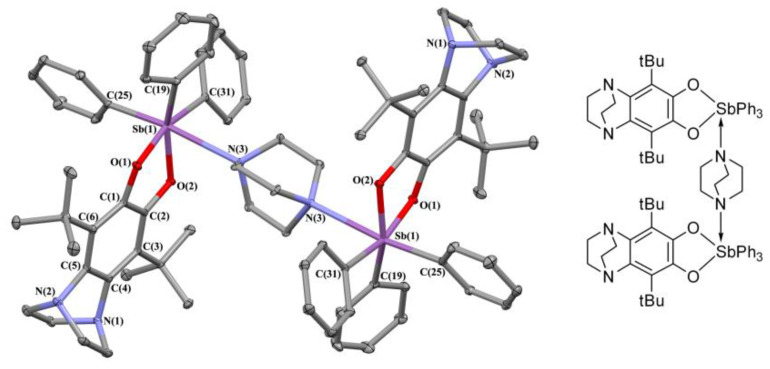
The X-ray structure of **10**. The hydrogen atoms are omitted. The selected bond lengths (Ǻ) and angles (°): Sb(1)-O(1) 2.0417(12), Sb(1)-O(2) 2.0124(12), Sb(1)-N(1) 2.727(2), Sb(1)-C(19) 2.1364(18), Sb(1)-C(25) 2.1355(18), Sb(1)-C(31) 2.1520(17), O(1)-C(1) 1.360(2), O(2)-C(2) 1.363(2), O(1)-Sb(1)-O(2) 78.72(5), O(2)-Sb(1)-C(19) 157.00(6), O(1)-Sb(1)-C(31) 165.97(6), N(3)-Sb(1)-C(25) 173.20(7).

**Figure 7:**
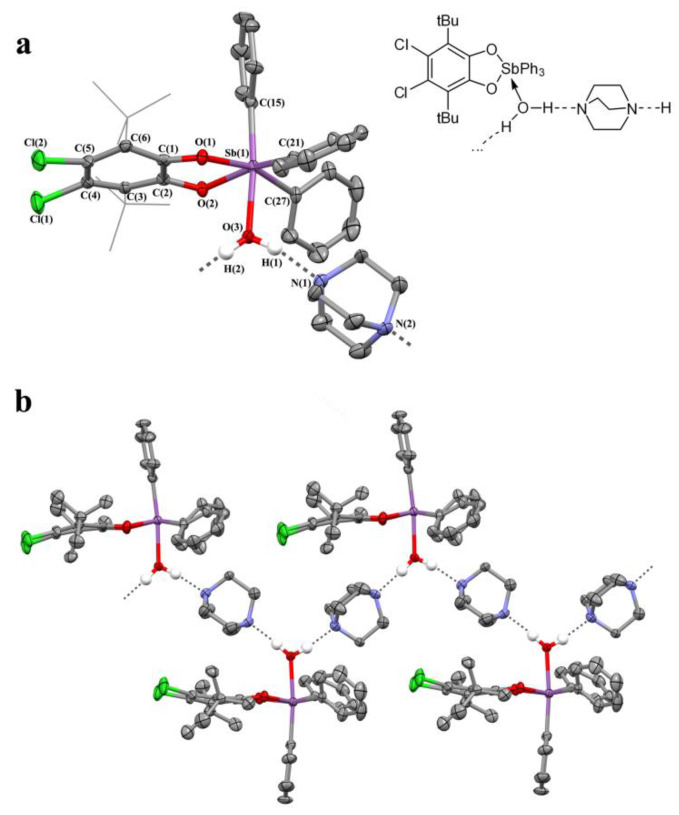
The X-ray structure of **12**: (**a**) the unit (4,5-Cl_2_-3,6-Cat)SbPh_3_∙H_2_O∙DABCO; (**b**) the 1D chain in crystal of **12**. The hydrogen atoms (except H_2_O) are omitted. The selected bond lengths (Ǻ) and angles (°): Sb(1)-O(1) 2.049(3), Sb(1)-O(2) 2.046(3), Sb(1)-O(3) 2.309(3), Sb(1)-C(15) 2.151(5), Sb(1)-C(21) 2.147(5), Sb(1)-C(27) 2.156(5), O(1)-C(1) 1.329(6), O(2)-C(2) 1.327(5), O(3)-H(1) 0.942(10), O(3)-H(2) 0.945(10), H(1)-N(1) 1.819(10), H(2)-N(2) 1.811(10), O(1)-Sb(1)-O(2) 77.50(12), O(2)-Sb(1)-C(21) 158.30(16), O(1)-Sb(1)-C(27) 162.34(17), C(15)-Sb(1)-O(3) 174.26(15).

**Figure 8:**
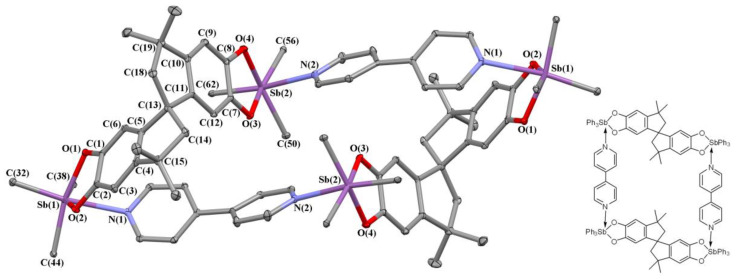
The X-ray structure of **13**. Thermal ellipsoids of 30% probability. The hydrogen atoms and carbons of phenyl groups (except atoms C(32), C(38), C(44), C(50), C(56), C(62)) are omitted.

The crystal structures of complexes [(3,6-DBCat)SbPh_3_]_2_·DABCO (**1**), [(3,6-DBCat)SbPh_3_]_2_·(Bipy) (**3**), [(3,6-DBCat)SbPh_3_]_2_·DABCO (**4**), [(3,5-DBCat)SbPh_3_]_2_·(Bipy) (**5**), [(4,5-(MeO)_2_-3,6-DBCat)SbPh_3_]_2_·(Bipy) (**8**), [(4,5-Piperaz-3,6-DBCat)SbPh_3_]_2_·DABCO (**10**), {[(4,5-Cl_2_-3,6-DBCat)SbPh_3_·H_2_O]·DABCO}_n_ (**12**), and {Ph_3_Sb(Cat-Spiro-Cat)SbPh_3_∙(Bipy)}_2_ (**13**) were determined by means of single-crystal X-ray analysis (Figure 1, Figure 2, Figure 3, Figure 4, Figure 5, Figure 6, Figure 7 and Figure 8, and Figures S27–S34 of ESI). The experimental and refinement details are given in Table S1 of Supplementary Materials. The selected bond distances and angles are given in Table S2 of Supplementary Materials. The mononuclear moieties (Cat)SbPh_3_ in molecules of **1**, **3**, **4**, **5**, and **10** are crystallographically identical. Each antimony atom possesses a distorted octahedral environment with redox active O,O’-chelating ligand and two phenyl groups in the base of octahedron. The nitrogen atom of neutral ligand and the third phenyl group are in the apical positions.

In all structures, the redox active ligand is in catecholato form. The O–C bonds in molecules of **1**, **3**, **4**, **5**, **8**, and **10** lie in the range of 1.357–1.366 Ǻ typical for ordinary O–C bonds in antimony catecholates [64,65,66,67,68,69,70], the six-membered carbon cycles are aromatic with the average C–C distances of 1.400, 1.401, 1.402, 1.402, 1.405, and 1.407 Ǻ, respectively. The metrical oxidation state (MOS) for complexes was calculated according to S.N. Brown [71] as −1.932, −1.959, −2.008, −1.971, −1.953, −1.900, and −1.904 (for **1**, **3**, **4**, **5**, **8**, and **10** respectively), confirming the dianionic nature of O,O’-chelating ligands. The Sb(1)–O(1) and Sb(1)–O(2) bond lengths in complexes are typical for antimony(V) catecholates, the equatorial Sb–C_Ph_ bonds are longer than the apical ones, which is also characteristic of such compounds [59,72]. The distances between antimony and nitrogen atoms differ in the range 2.494(2)–2.773(5) Å. This is somewhat larger than the sum of the covalent radii of Sb and N in the octahedral geometry (1.43 + 0.74 = 2.17 Å [73]; 1.39 + 0.71 = 2.10 Å [74]), but significantly less than the sum of their van der Waals radii (2.2 + 1.6 = 3.8 Å [75]; 2.06 + 1.55 = 3.61 Å [76]), thus pointing to the donor–acceptor nature of the interaction. These bonds in our complexes lie in the wide range of Sb–N bonds of donor–acceptor nature observed in many different antimony compounds [77]. Thus, all structures have some common features, but they also have certain differences.

The molecule of **1** is centrosymmetric, and the mononuclear fragments (Cat)SbPh_3_ are rotated relative to each other by 180° and the catecholate planes are parallel to each other (Figure S27 of ESI). The intramolecular distance between two antimony atoms in molecule of **1** is 8.236 Å, and between planes of π-systems of two catecholate ligands is 7.372 Å. The angle between catecholate and pyrazine planes is 54.2°. The antimony atom Sb(1) leaves the plane of the octahedron base formed by O(1), O(2), C(21), and C(27) atoms by 0.35 Å. Catecholate planes are slightly distorted, the bent angle of chelate ring along O...O is 5.67°, and the Sb(1) lies 0.085 Å out the catecholate plane C_6_O_2_. The torsion angle O(1)C(1)C(2)O(2) is 0.80° only.

In general, the molecular structures of 4,4′-bipyridine linked complexes **3** (Figure 2) and **5** (Figure 4) are close. The distances of Sb(1) atom from an octahedron basal plane and from the catecholate π-system plane C_6_O_2_ are 0.32 and 0.04 Å for **3**, 0.28 and 0.13 Å for **5**, respectively. The bent angle of chelate ring along O...O is 5.94° in **5** but close to 0 in **3**, and chelate rings are slightly distorted: the torsion angles O(1)C(1)C(2)O(2) are 2.28° (in **5**) and 2.68° (in **3**). The Bipy ligand is planar in both complexes. In addition, catecholates are parallel to each other and, as in **1**, they are oppositely directed (180°) (Figures S28 and S30 of ESI). The Py part of Bipy is located relative to the catecholato plane at an angle of 62.08° in **3** and 57.61° in **5**.

In contrast, the structure of 4,4′-bipyridine-linked complex **8** (Figure 5) differs remarkably. The chelate rings of Sb(1) and Sb(2) atoms are more distorted: the bent angle of chelate ring along O(1)...O(2) is 9.38° and O(5)...O(6) is 10.28°; the corresponding torsion angles OCCO are 1.07 and 3.43°; the deviation of Sb(1) atom from an octahedron base is 0.280 Å, and from the catecholato plane is 0.241 Å; for Sb(2) these values are 0.318 and 0.273 Å, respectively. The catecholate planes are not parallel, the corresponding angle is 19.13° (Figure S31 of ESI). In contrast to **1**, **3**, and **5**, the mononuclear fragments (Cat)SbPh_3_ in **8** are rotated relative to each other by 111°. The angle between pyridine planes in Bipy ligand in **8** is 35.55°, and the angles between pyridine planes and catecholato planes are 61.65° and 74.84° for ligands environment at Sb(a) and Sb(2) atoms, respectively.

The same situation was found for DABCO-linked complex **4** based on 3,6-di-tert-butyl-catecholate (Figure 3). Catecholato planes are not parallel with an angle of 18.43°, mononuclear fragments (Cat)SbPh_3_ are rotated relative to each other by 86° (Figure S29 of ESI). The bent angle along O(1)...O(2) line in chelate cycle is 17.28°, and the central antimony atom Sb(1) deviates 0.478 Å from the plane of catecholato ligand—the highest value in this series. The torsion angle O(1)C(1)C(2)O(2) in 4 is 0.34° only.

The related complex **10** (with piperazine group in the 4th and 5th positions of 3,6-di-tert-butyl-catecholate ligand) has a more distorted Cat ligand (Figure 6, and Figure S32 of ESI): the six-membered carbon cycle is folded according to the principle of a propeller blade. The torsion angle O(1)C(1)C(2)O(2) is 5.59°, and the bent angle of chelate ring along O(1)...O(1) line has a value of 18.05° maximal in this series of complexes. The distances “Sb(1)... base of octahedron” and “Sb(1)... Cat plane” are 0.250 and 0.444 Å, respectively.

Complex **12** has rather different structure. In solid state, molecules of (4,5-Cl_2_-3,6-Cat)SbPh_3_∙H_2_O and DABCO form 1D polymeric chains via the intermolecular hydrogen bonding of water and DABCO molecules (Figure 7, Figure S33 of ESI).

The chelating catecholate ligand in **12** is strongly distorted: the six-membered carbon cycle C(1–6) is twisted along C(3)...C(6) line, and the angle between planes C(1,2,3,6) and C(3,4,5,6) is 12.6(2)°. At the same time, the torsion angle O(1)–C(1)–C(2)–O(2) is 2.2(5)°, and the deviation of Sb(1) atom from the basal plane of the octahedron is 0.263 Ǻ. The intermolecular contacts O(3)–H(1)...N(1) and O(3)–H(2)...N(2) of water molecule with two neighboring DABCO molecules are 1.819(10) and 1.811(10) Å, respectively.

The tetranuclear molecule of **13** is centrosymmetrical in crystal. In **13** (Figure 8; Figure S34 of ESI), two binuclear molecules Ph_3_Sb(Cat-Spiro-Cat)SbPh_3_ are connected by two 4,4′-bipyridine molecules via the donor–acceptor interactions Sb(1)–N(1) and Sb(2)–N(2) (2.439(3) and 2.463(3) Å, respectively).

Antimony atoms Sb(1) and Sb(2) in **13** dispose in a distorted octahedral environment. The chelate cycles at both antimony atoms are not planar: the bent angle of chelate cycle Sb(1)O(1)C(1)C(2)O(2) along O(1)...O(2) line is 13.50°, and the bent angle of chelate cycle Sb(2)O(3)C(7)C(8)O(4) along O(3)...O(4) line is 14.82°. The torsion angles O(1)C(1)C(2)O(2) and O(3)C(7)C(8)O(4) are 0.05 and 2.05°, respectively. The deviations of antimony atoms Sb(1) and Sb(2) from the octahedron basal planes are close (Sb(1)... plane O(1)O(2)C(38)C(44) is 0.269 Å, and Sb(2)... plane O(3)O(4)C(50)C(56) is 0.254 Å). The metrical oxidation state (MOS) [71] of O,O’-chelating ligands in complex **13** was calculated to be −2.040 (Cat1, Figure S34 of ESI) and −2.064 (Cat2, Figure S34 of ESI), which are the biggest values in the series of complexes in this work.

### 2.3. Charge Density in 5_ED_

**Figure 9:**
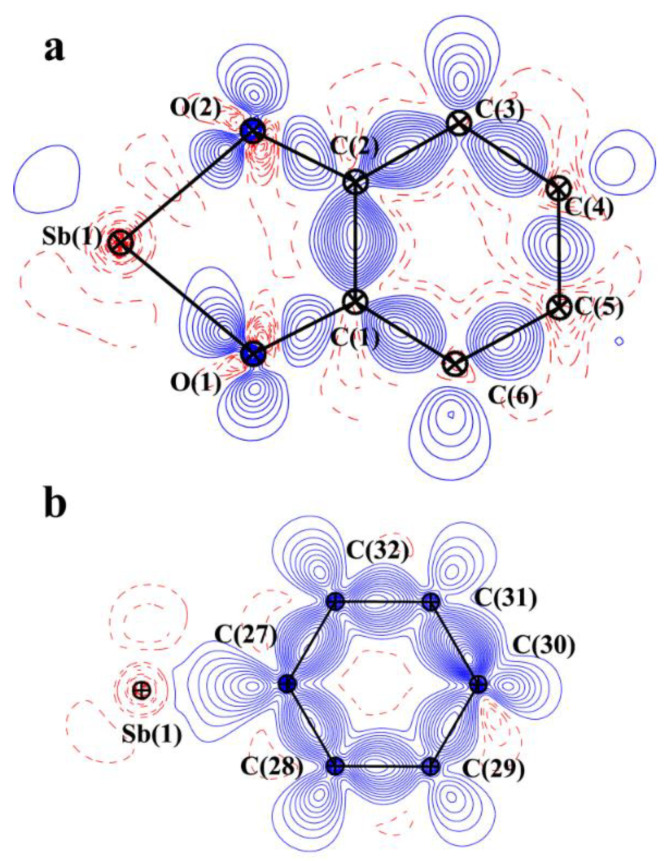
Experimental DED sections in **5_ED_** (contour ± 0.05 e/Å^3^): the plane of the Sb(1)O(1)O(2) fragment (**a**); the plane of the Ph ligand (**b**). The solid lines (blue) show the concentration of DED, whereas the dashed lines (red) show the depletion of DED. H atoms are omitted for clarity.

Due to the fact that crystals of complex **5** (hereinafter referred to as **5_ED_**) had a high reflectivity, we carried out a high resolution X-ray diffraction experiment to investigate the electron density distribution in this complex. Details of multipole refinement are given in Supplementary Materials (Figures S35–S37 of ESI). The distribution of the deformation electron density (DED) around the Sb atom in **5_ED_** has a clearly pronounced polar (probably ionic) character (Figure 9a,b).

**Figure 10:**
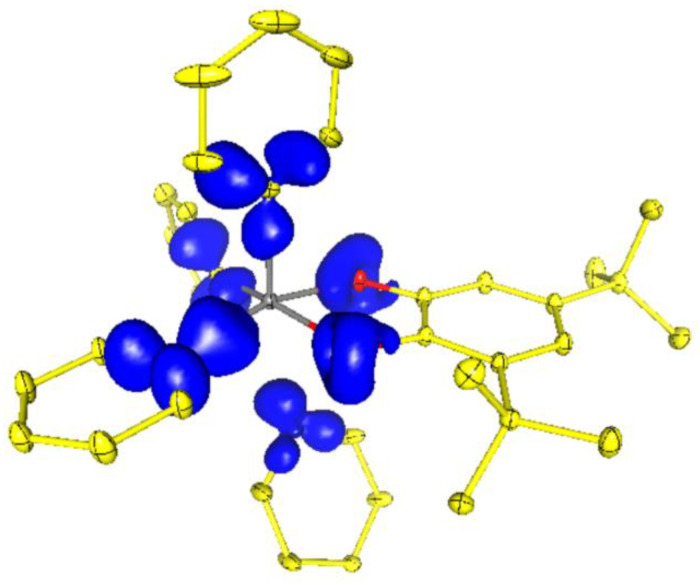
DED isosurface (0.1 a.u.) in the asymmetric unit of **5_ED_**.

The DED maxima on the Sb–O and Sb–C bonds are markedly shifted toward the oxygen and carbon atoms, respectively. The DED maxima on the O–C bonds are located at the middle of the corresponding distances in **5_ED_**, whereas in the related catecholate Sb(V) complexes [78], the maxima are shifted toward the oxygen atoms. The angle between the lone electron pairs of oxygen atoms and the Sb–O and O–C bonds is close to 120°. The lone electron pair of the nitrogen atom of the 4,4′-dipyridyl molecule is directed away from the antimony atom (Figure 10) towards approximately the middle of the Sb(1)–C(15) bond (Figure S38 of ESI). A similar situation is observed in complexes **1** and **3**.

To understand the nature of the chemical bonds in **5_ED_** (Table 1), we used Bader’s theory [79]. The corresponding calculations have shown that the Sb–O, Sb–N, and Sb–C bonds in **5_ED_** are intermediate interactions (∇^2^*ρ*(r) > 0, *h*_e_(r) < 0; covalent polar bonds), whereas the O–C, N–C, and C–C bonds are shared (2(r) < 0, he(r) < 0; covalent bonds). A similar situation is observed in related catecholate complexes (Ph_3_(4,5-OMe-3,6-tBu-Cat)Sb∙MeCN and Ph_3_(4,5-N_2_C_4_H_6_-3,6-tBu-Cat)Sb∙MeOH) [78]. Using the Espinosa–Molins–Lecomte correlation [80], we estimated the energy of the donor–acceptor interaction between the antimony(V) and nitrogen atom of the 4,4′-dipyridyl molecule. The energy of this interaction is 7.5 kcal/mol.

The analysis of charges obtained by electron density integration inside the atomic basin has revealed that the charge on antimony atom is 2.07 e. The charges on phenyls and catecholate substituents and also 4,4′-dipyridyl molecule are −0.4 ÷ −0.53 e, −0.41 and −0.32 e, respectively. Thus, the bridging 4,4′-dipyridyl molecule is negatively charged and apparently can coordinate on itself in complex **5_ED_** small electrophilic molecules, which potentially can undergo unusual transformations in the coordination sphere of antimony atom.

**Figure 11:**
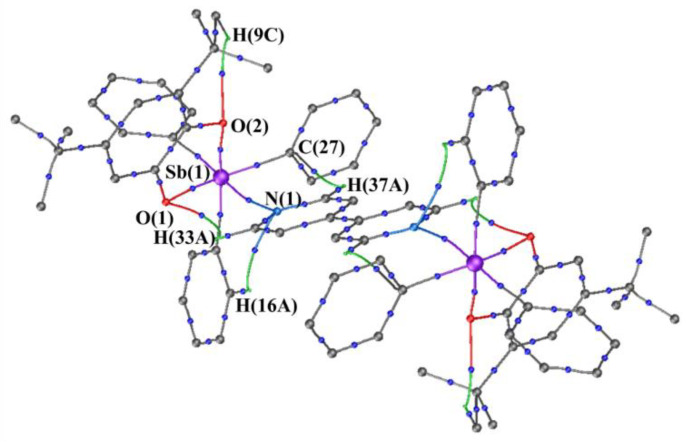
Experimental molecular graph of the complex **5_ED_**. The hydrogen atoms, except those participating in intramolecular interaction, are omitted for clarity. Only critical points (3,–1) (blue color) are presented for clarity.

Analysis of the experimental molecular graph has shown that the intra-molecular O...H, N...H and C...H interactions are realized between the catecholate part of the complex and the 4,4′-dipyridyl molecule (Figure 11). The total energy of these interactions is ~6 kcal/mol and is comparable with the Sb(1)–N(1) bonding energy. Perhaps these interactions are responsible for the direction of lone pair of the nitrogen atoms towards the middle of the Sb(1)–C(15) bond (Figure S38 of ESI).

### 2.4. Cyclic Voltammetry

The cyclic voltammetry can provide an additional information about the electronic structure and the nature of interactions between different ligands in a complex molecule. The binuclear antimony(V) complexes with bidentate ligands **1**–**7**, **9**, and tetranuclear antimony(V) complex **13** were investigated by means of CV. The electrochemical potentials of these catecholates are given in Table 2.

**Figure 12:**
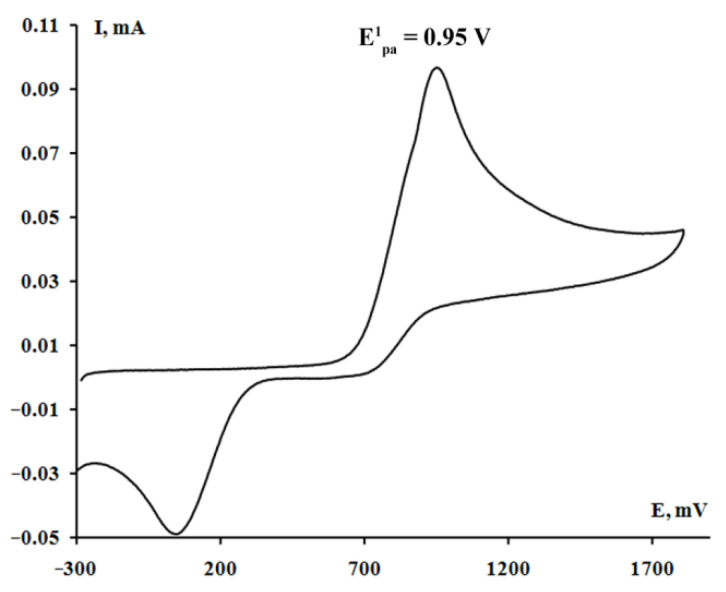
CV of complex **5** in the sweep range of −0.3 to 1.8 V (CH_2_Cl_2_, GC, C = 3 × 10^−3^ M, 0.1 M Bu_4_NClO_4_, vs. Ag/AgCl/KCl(sat.)).

The electrochemical behavior of the complexes depends on the coordinated N-donor ligand. Conventionally, these complexes can be divided into two groups: the first one contains Pyr, Bipy, PySSPy (complexes **1**–**3**, **5**–**7**, **13**), and the second one—complexes **4** and **9** with DABCO. The coordination of pyrazine, Bipy, and PySSPy at the antimony atom changes their mechanism of electrooxidation: instead of two successive redox stages Cat/SQ and SQ/Cat, one multielectron stage is observed (Figure 12). Previously, a similar effect was observed for mononuclear triphenylantimony(V) catecholates, containing coordinated pyridine molecules [62]. In the case of pyrazine, the potential value coincides with that for the complex with pyridine. For complexes with bridging Bipy and PySSPy (complexes **2**, **3**, **5**, **7**), there is an insignificant shift of the peaks to the anodic region (0.95–1.05 V).

The two-electron oxidation of the catecholate group results in the formation of o-benzoquinone and its subsequent de-coordination. A quasi-reversible reduction peak of the de-coordinated quinone is fixed on the reverse branch of the CV during a pulsed potential sweep.

**Figure 13:**
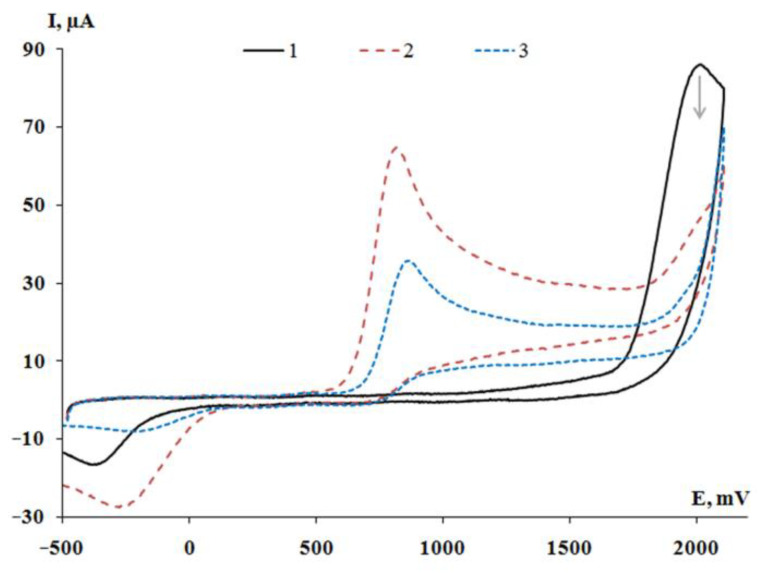
The CV of free PySSPy (curve 1); The CV of reaction mixture of (3,6-DBCat)SbPh_3_ and PySSPy in a ratio 1:1 (curve 2); The CV of a reaction mixture of (3,6-DBCat)SbPh_3_ and PySSPy in a ratio 2:1 (curve 3). The potential sweep from −0.5 to 2.1 V, CH_2_Cl_2_, GC, C = 3 × 10^−3^ M, 0.1 M Bu_4_NClO_4_, vs. Ag/AgCl/KCl(sat.).

We have carried out the model CV experiments with free PySSPy and an antimony complex (3,6-DBCat)SbPh_3_ to confirm the coordination of pyridine-containing ligands in a solution. The addition of triphenylantimony(V) 3,6-di-tert-butylcatecholate to a solution of PySSPy in a molar ratio of 1:1 leads to a significant decrease in the intensity of the free ligand oxidation wave (E_p_ = 1.98 V), while in a ratio of 2:1— to its disappearance. In this case, an oxidation wave of complex **2** appears (Figure 13). Oxidation waves of (3,6-DBCat)SbPh_3_ (at E^1^_1/2_ = 0.89 V, E^2^_p_ = 1.40 V vs. Ag/AgCl/KCl(sat.) [81]) are not observed on the CV curve.

**Figure 14:**
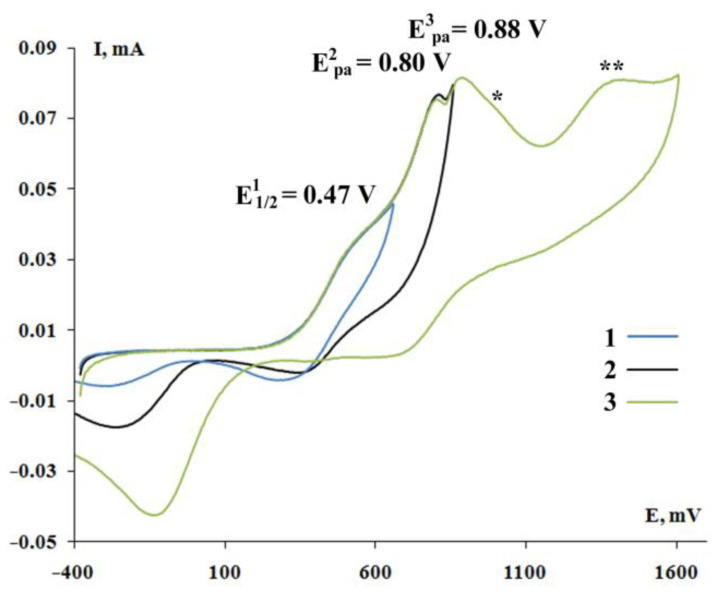
The CV of complex [(3,5-DBCat)SbPh_3_]_2_·DABCO (**9**) at the potential sweep from −0.4 to 0.65 V (curve 1); from −0.4 to 0.85 V (curve 2); from −0.4 to 1.6 V (curve 3) (CH_2_Cl_2_, GC, C = 3 × 10^−3^ M, 0.1 M Bu_4_NClO_4_, Ag/AgCl/KCl(sat.)). * and **—the oxidation peaks of mononuclear (3,5-DBCat)SbPh_3_ (E^1^_1/2_ = 0.91 V, E^2^_p_ = 1.40 V vs. Ag/AgCl/KCl(sat.)).

The coordination of the DABCO ligand is accompanied by a significant shift in the oxidation potentials of the catecholate ligand to the cathodic region (by 0.4 V), compared to the initial complex. Such a significant shift of the oxidation potentials to the cathodic region, in comparison with the initial catecholate is explained by a significant increase in the total electron density of the six-coordination metal site upon coordination of the donor diazabicyclooctane. The observed effect on the values of the oxidation potentials of the complexes is similar to the effect of coordination over the antimony atom of charged nucleophilic agents (bromide anion, hydroxy group) [61]. The CVs of DABCO containing complexes **4** and **9** (Figure 14) contain three oxidation waves at the potential range to 1.30 V. The first oxidation is quasi-reversible and may be assigned to the oxidation process Cat/SQ. As compared with the initial complexes, the current ratio I_c_/I_a_ decreases to 0.7, pointing out the lower stability of the electrogenerated particles. Moreover, the reverse branch of CV shows the appearance of the reduction peak of the product of the chemical stage following the electron transfer. The second irreversible redox transition we assign to the stage of the oxidation of a coordinated o-benzosemiquinone to o-benzoquinone.

However, the additional peaks observed in CV indicate a more complex mechanism of electrotransformations of the complexes. Suppose the complex was destroyed after the first one-electron oxidation. In that case, a second oxidation peak could be observed at a potential characteristic of (3,6-DBCat)SbPh_3_, but this value is also shifted to the cathodic region. Based on potential values of 0.88 and 0.91, these peaks can correspond to free antimony catecholate complexes not bound to DABCO or de-coordinated nitrogen-containing ligand (0.90 V). With an increase in the potential sweep to 1.7 V, it is possible to fix a peak at 1.40 V, which is characteristic of the second redox transition [Ph_3_Sb(3.6-DBSQ)]^+^/[Ph_3_Sb(3.6-DBBQ)]^2+^ of mononuclear complex without N-donor ligand [81]. One can assume that during the CV experiment, the binuclear complex decomposes as a result of the initial oxidation of the complex with DABCO. This decomposition accompanied by the de-coordination of one of the catecholate fragments of (3,6-DBCat)SbPh_3_ and the release of DABCO.

The CV of complex **13** shows two stages of oxidation. The coordination of the bipyridine ligand leads to an insignificant shift of the oxidation potential to the cathodic region (0.83 V) relative to the initial binuclear derivative (0.85 V) [58]. In this case, we do not fix the separation of two oxidation stages; just as in the case of pyridine coordination, a multielectron process occurs, which leads to the oxidation of catecholate groups and subsequent possible elimination of the Ph_3_Sb^2+^ dication and the formation of a quinone–catecholate complex. The absence of separation of the second stage into two peaks indirectly testifies to the preservation of the coordination of the bridging nitrogen-containing ligand.

## 3. Materials and Methods

### 3.1. General

The synthesis, isolation, and study of the properties of the complexes were carried out in evacuated ampoules in the absence of oxygen. The organic solvents used in work were purified according to standard procedures [82]. Initial complexes triphenylantimony(V) 3,6-di-tert-butyl- and 3,5-di-tert-butyl-catecholates [83], 4,5-(N,N′-diethylenediamine-1,4-diyl)-3,6-di-tert-butylcatecholate [50], 4,5-dichloro-3,6-di-tert-butyl-catecholate [62], 4,5-dimethoxy-3,6-di-tert-butyl-catecholate [49], bis-triphenylantimony(V) spiro-bis-catecholate [58] were synthesized by the known methods.

The IR spectra were recorded on an FSM-1201 FT-IR spectrometer in KBr pellets.

The NMR spectra were registered using Bruker Avance DPX-200 (200 MHz) and Bruker AVANCE New (300 MHz) spectrometers with TMS as an internal standard and CDCl_3_ as a solvent. The chemical shift values are given in ppm with the reference to solvent and the coupling constants (J) are given in Hz. The elemental analysis (C, H) was carried out on a Euro EA 3000 elemental analyzer, as well as (Sb) by a pyrolytic decomposition in an oxygen flow.

Electrochemical studies were carried out using VERSASTAT-3 potentiostate (PAR) in three-electrode mode. The stationary glassy carbon (d = 2 mm) disk was used as working electrode; the auxiliary electrode was a platinum-flag electrode. The reference electrode was Ag/AgCl/KCl (sat.) with watertight diaphragm. All measurements were carried out under argon. The samples were dissolved in the pre-deaerated solvent. The scan rate was 0.2 V∙s^−1^. The supporting electrolyte 0.1 M Bu_4_NClO_4_ (99%, electrochemical grade, Fluka) was dried in vacuum (48 h) at 50 °C.

### 3.2. The X-ray Diffraction

The X-ray diffraction data were collected on a Bruker Smart Apex I (**1**, **8**), Oxford Xcalibur Eos (**3**–**5**, **12**) and Bruker D8 Quest (**10**, **13**) diffractometers (Mo-K_α_ radiation, ω-scan technique, λ = 0.71073 Å). The intensity data were integrated by SAINT (**1**, **8**, **10**, **13**) [84] and CrysAlisPro (**3**–**5**, **12**) [85] programs. SADABS program (**1**, **8**, **10**, **13**) [86] and SCALE3 ABSPACK algorithm (**3**-**5**, **12**) [85] were used to perform absorption corrections. All structures were solved by dual method [87] and refined on $F_{hkl}^{2}$ using SHELXTL package [88]. All non-hydrogen atoms were refined anisotropically. The hydrogen atoms were placed in calculated positions and were refined in the riding model (U_iso_(H) = 1.5 U_eq_(C) for CH_3_-groups and U_iso_(H) = 1.2 U_eq_(C) for other groups). The main crystallographic data and structure refinement details for all complexes are presented in Table S1. CCDC 2174897 (**1**), 2174898 (**3**), 2174899 (**4**∙n-hexane), 2174900 (**5**; low resolution-IAM), 2174901 (**5**; high resolution-IAM), 2174902 (**5**; high resolution multipole refinement), 2174903 (**8**∙toluene), 2174904 (**10∙**3.5toluene), 2174905 (**12∙**0.25toluene), and 2174906 (**13**∙6toluene) contain the supplementary crystallographic data. These data can also be obtained free of charge at ccdc.cam.ac.uk/structures/ (accessed on 19 September 2022) from the Cambridge Crystallographic Data Centre.

### 3.3. Synthesis and Characterization

#### 3.3.1. The General Synthetic Method of Binuclear Triphenylantimony(V) Complexes with Linker N-Donor Ligands

The toluene solution of the corresponding starting triphenylantimony(V) catecholate (0.5 mmol, 40 mL) was slowly added with a stirring to the toluene solution of N-donor ligand (0.25 mmol, 30 mL). The stirring was continued for 1 h at temperatures of 40–50°C. The toluene was completely removed under reduced pressure, and the formed residue was recrystallized from different solvents depending on complex: a toluene/*n*-hexane mixture (1:2) for complexes **1**, **2** and **12**; toluene/*n*-hexane mixture (1:1) for complexes **6**, **7**, **8**, **11**; toluene for complexes **3**, **5**, **10** and **13**; and n-hexane for complexes **4** and **9**.

#### 3.3.2. [(3,6-. DBCat)SbPh_3_]_2_(Pyr) (**1**)

The light orange crystalline powder of **1** was isolated from a toluene–n-hexane (1:2) mixture. Yield is 87%. Calcd. for C_68_H_74_N_2_O_4_Sb_2_ (%): C, 66.57; H, 6.08; Sb, 19.85. Found (%): C, 66.64; H, 6.11; Sb, 19.98. IR (nujol, cm^−1^): 1576 w (ν arom. C–N); 1483 s, 1436 m (ν arom. C–C); 1400 s, 1353 m, 1331 w, 1308 m, 1282 m, 1265 m, 1241 s (ν C–O), 1205 w, 1182 w, 1145 m, 1074 m, 1062 m, 1027 m, 986 s, 978 s, 943 m, 929 w, 810 m; 795 s, 729 s, 692 s (δ SbPh_3_); 664 w, 458 s (ν Sb–O). ^1^H NMR (CDCl_3_, δ, ppm): 1.43 (s, 36H, tBu), 6.62 (s, 4 H, arom. C_6_H_2_), 7.40–7.55 (m, 18 H, *o,p*-H, Ph), 7.73–7.87 (m, 12 H, *m*-H, Ph), 8.54 (s, 4 H, pyrazine). ^13^C{^1^H} NMR (CDCl_3_, δ, ppm): 29.59, 34.15, 114.30, 129.11, 131.03, 132.14, 135.03, 138.20, 145.14, 145.30.

#### 3.3.3. [(3,6-. DBCat)SbPh_3_]_2_·(PySSPy) (**2**)

The orange powder was isolated from a toluene–n-hexane (1:2) mixture. Yield is 78%. Calcd. for C_74_H_78_N_2_O_4_ S_2_Sb_2_ (%): C, 65.00; H, 5.71; Sb, 17.86. Found (%): C, 65.24; H, 5.81; Sb, 17.68. IR (nujol, cm^−1^): 1578 s, 1542 m (ν arom. C–N); 1479 s, 1430 s (ν arom. C–C); 1406 s, 1381 s, 1373 s, 1355 m, 1348 m, 1310 m, 1285 m, 1266 s, 1242 s (ν C–O), 1216 m, 1205 m, 1183 m, 1148 m, 1072 s, 1063 s, 1023 m, 1003 w, 996 w, 980 s, 943 s, 852 w, 810 s, 800 m; 791 m, 733 s, 695 s (δ SbPh_3_); 661 w, 646 s, 603 w, 550 w, 492 m, 458 s (ν Sb–O). ^1^H NMR (CDCl_3_, δ, ppm): 1.43 (s, 36 H, tBu), 6.61 (s, 4 H, arom. C_6_H_2_), 7.28–7.35 (m, 4 H, PySSPy), 7.40–7.55 (m, 18 H, *o,p*-H, Ph), 7.74–7.86 (m, 12 H, *m*-H, Ph), 8.41–8.50 (m, 4 H, PySSPy). ^13^C{^1^H} NMR (CDCl_3_, δ, ppm): 29.58, 34.14, 114.23, 120.01, 129.04, 130.89, 132.13, 135.01, 138.66, 145.36, 146.70, 149.78.

#### 3.3.4. [(3,6-. DBCat)SbPh_3_]_2_·(Bipy) (**3**)

Orange crystals were isolated from toluene solution. Yield is 88%. Calcd. for C_74_H_78_O_4_N_2_Sb_2_ (%): C, 67.61; H, 6.10; Sb, 19.09. Found (%): C, 67.89; H, 5.99; Sb, 19.31. IR (nujol, cm^−1^): 1624 w, 1603 s, 1576 m (ν arom. C–N); 1532 m, 1479 s, 1431 s (ν arom. C–C); 1401 s, 1354 m, 1314 s, 1304 m, 1279 m, 1257 s, 1241 s (ν C–O), 1234 s, 1215 m, 1201 w, 1183 m, 1147 m, 1097 w, 1077 s, 1063 s, 1022 m, 997 s, 977 s, 940 s, 924 w, 862 w, 850 w, 807 s; 788 m, 734 s, 694 s (δ SbPh_3_); 660 w, 645 m, 616 m, 601 w, 570 w, 548 w, 516 w, 455 s (ν Sb–O). ^1^H NMR (CDCl_3_, δ, ppm): 1.43 (s, 36 H, tBu), 6.61 (s, 4 H, arom. C_6_H_2_), 7.40–7.55 (m, 22 H: 18 H, *o,p*-H, Ph + 4 H, Bipy), 7.72–7.85 (m, 12 H, *m*-H, Ph), 8.68–8.80 (m, 4H, Bipy). ^13^C{^1^H} NMR (CDCl_3_, δ, ppm): 29.59, 34.15, 114.27, 121.40, 129.08, 130.96, 132.15, 135.03, 138.43, 145.34, 150.65.

#### 3.3.5. [(3,6-. DBCat)SbPh_3_]_2_·(DABCO) (**4**)

Orange crystalline product was isolated from n-hexane. Yield is 73%. Calcd. for C_70_H_82_N_2_O_4_Sb_2_ (%): C, 66.72; H, 6.57; Sb, 19.34. Found (%): C, 66.79; H, 6.61; Sb, 19.38. IR (nujol, cm^−1^): 1402 m (ν arom. C–C), 1350 s, 1305 m, 1284 m, 1261 m, 1240 s (ν C–O), 1206 m, 1183 w, 1160 w, 1148 m, 1074 m, 1063 m, 1051 m, 1023 m, 995 m, 975 m, 939 m, 925 m, 834 w, 809 m; 792 m, 773 m, 730 s, 693 s (δ SbPh_3_); 659 m, 645 m, 599 w, 548 w, 517 w, 460 s (ν Sb–O). ^1^H NMR (CDCl_3_, δ, ppm): 1.43 (s, 36H, tBu), 2.79 (s, 12H, CH_2_), 6.62 (s, 4 H, C_6_H_2_), 7.40–7.55 (m, 18 H, *o,p*-H, Ph), 7.73–7.86 (m, 12 H, *m*-H, Ph). ^13^C{^1^H} NMR (CDCl_3_, δ, ppm): 29.58, 34.14, 47.37, 114.30, 129.10, 131.03, 132.14, 135.03, 138.17, 145.30.

#### 3.3.6. [(3,5-. DBCat)SbPh_3_]_2_·(Bipy) (**5**)

Orange crystals were isolated from toluene solution. Yield is 85%. Calcd. for C_74_H_78_O_4_N_2_Sb_2_ (%): C, 67.61; H, 6.10; Sb, 19.09. Found (%): C, 67.91; H, 5.97; Sb, 19.37. IR (nujol, cm^−1^): 1532 m (ν arom. C–N), 1432 s, 1418 s (ν arom. C–C); 1405 s, 1356 m, 1320 s, 1281 s, 1263 m, 1244 s (ν C–O), 1220 m, 1210 m, 1183 m, 1160 w, 1110 w, 1069 s, 1023 m, 994 m, 982 s, 915 w, 849 m, 828 m, 809 s; 752 m, 736 s, 695 s (δ SbPh_3_); 652 m, 617 s, 537 w, 514 w, 480 w; 462 s, 453 s (ν Sb–O). ^1^H NMR (CDCl_3_, δ, ppm): 1.29 (s, 18H, tBu), 1.46 (s, 18H, tBu), 6.70 (d, ^4^J(H,H) = 2.2 Hz, 2 H, arom. C_6_H_2_), 6.96 (d, ^4^J(H,H) = 2.2 Hz, 2 H, arom. C_6_H_2_), 7.40–7.65 (m, 22 H: 18 H, *o,p*-H, 6 Ph + 4 H, Bipy), 7.70–7.86 (m,12 H, *m*-H, 3 Ph), 8.70–8.80 (m, 4H, Bipy). ^13^C{^1^H} NMR (CDCl_3_, δ, ppm): 29.74, 31.79, 34.44, 34.67, 107.84, 112.46, 121.40, 129.13, 129.58, 131.01, 131.73, 133.21, 134.12, 135.08, 138.23, 139.56, 150.60.

#### 3.3.7. [(4,5-. pip-3,6-DBCat)SbPh_3_]_2_·(Bipy) (**6**)

A fine-crystalline orange product was isolated from a toluene–n-hexane (1:1) mixture. Yield is 83%. Calcd. for C_82_H_90_N_6_O_4_Sb _2_ (%): C, 67.13; H, 6.18; Sb, 16.60. Found (%): C, 67.30; H, 6.29; Sb, 16.75. IR (nujol, cm^−1^): 1576 m, 1532 m (ν arom. C–N); 1477 s, 1431 s (ν arom. C–C); 1350 m, 1324 s, 1310 m, 1289 m, 1267 m, 1238 s (ν C–O), 1223 s, 1205 m, 1180 m, 1177 m, 1077 m, 1059 m, 1052 m, 1022 m, 997 s, 977 s, 940 s, 924 w, 862 w, 850 w, 807 s; 788 m, 734 s, 694 s (δ SbPh_3_); 660 w, 645 m, 616 m, 601 w, 570 w, 563 m, 519 m, 465 s (ν Sb–O). ^1^H NMR (CDCl_3_, δ, ppm): 1.61 (s, 36 H, tBu), 2.56–2.78 (m, 8H, CH_2_, N(CH_2_CH_2_)_2_N), 2.82–3.10 (m, 8H, CH_2_, N(CH_2_CH_2_)_2_N), 7.40–7.60 (m, 22 H: 18 H, *o, p*-H, 6 Ph + 4H, Bipy), 7.70–7.82 (m, 12 H, *m*-H, 6 Ph), 8.70–8.78 (m, 4H, Bipy). ^13^C{^1^H} NMR (CDCl_3_, δ, ppm): 32.34, 35.47, 50.16, 121.38, 126.48, 128.22, 129.00, 130.89, 135.08, 138.27, 141.19, 145.56, 150.68.

#### 3.3.8. [(4,5-. Cl_2_-3,6-DBCat)SbPh_3_]_2_·(Bipy) (**7**)

A fine-crystalline orange product was isolated from a toluene–n-hexane (1:1) mixture. Yield is 85%. Calcs. for C_74_H_74_ Cl_4_N_2_O_4_Sb_2_ (%): C, 61.69; H, 5.18; Sb, 16.90. Found (%): C, 61.58; H, 5.09; Sb, 16.77. IR (nujol, cm^−1^): 1536 m (ν arom. C–N), 1479 s, 1429 s, 1408 s (ν arom. C–C); 1302 m, 1265 m, 1247 s (ν C–O), 1218 s, 1204 s, 1182 m, 1159 m, 1064 s, 1030 m, 998 m, 972 s, 927 w, 838 s, 807 s; 775 s, 730 s, 693 s (δ SbPh_3_); 673 m, 659 m, 624 s, 590 w, 550 w, 497 s (ν Sb–O). ^1^H NMR (CDCl_3_, δ, ppm): 1.62 (s, 36H, tBu), 7.40–7.60 (m, 22 H: 18 H, *o, p*-H, 6 Ph + 4 H, Bipy), 7.66–7.76 (m, 12 H, *m*-H, 6 Ph), 8.62–8.70 (m, 4H, Bipy). ^13^C{^1^H} NMR (CDCl_3_, δ, ppm): 32.25, 38.52, 121.46, 123.62, 129.21, 129.74, 131.14, 134.13, 134.76, 137.93, 145.68, 150.38.

#### 3.3.9. [(4,5-(MeO)2-3,6-. DBCat)SbPh_3_]_2_·(Bipy) (**8**)

Orange crystals were isolated from a toluene–n-hexane (1:1) mixture. Yield is 81%. Calcd. for C_78_H_86_O_8_N_2_Sb_2_ (%): C, 65.83; H, 6.09; Sb, 17.11. Found (%): C, 65.91; H, 6.17; Sb, 17.27. IR (nujol, cm^−1^): 1536 w (ν arom. C–N); 1488 m, 1477 m, 1432 s (ν arom. C–C); 1354 m, 1302 w, 1272 m, 1225 m (ν C–O), 1220 m, 1196 w, 1182 w, 1158 w, 1108 m, 1060 s, 1022 w, 998 m, 979 m, 910 m, 879 m, 805 m, 773 w; 739 m, 734 s, 695 s (δ SbPh_3_); 654 w, 619 m, 574 w, 545 w, 520 w, 460 s, 453 s (ν Sb–O). ^1^H NMR (CDCl_3_, δ, ppm): 1.55 (s, 36 H, tBu), 3.7 (s, 12 H, OCH_3_), 7.38–7.58 (m, 22 H: 18 H, *o, p*-H, 6 Ph, + 4 H, Bipy), 7.70–7.82 (m, 12 H, *m*-H, 6 Ph), 8.68–8.76 (m, 4H, Bipy). ^13^C{^1^H} NMR (CDCl_3_, δ, ppm): 31.69, 36.17, 60.76, 121.38, 125.10, 128.22, 129.05, 130.93, 134.98, 138.35, 141.60, 145.11, 150.63.

#### 3.3.10. [(3,5-. DBCat)SbPh_3_]_2_·(DABCO) (**9**)

Orange crystals were isolated from n-hexane. Yield is 69%. Calcd. for Calcd. for C_70_H_82_N_2_O_4_Sb_2_ (%): C, 66.72; H, 6.57; Sb, 19.34. Found (%): C, 66.90; H, 6.69; Sb, 19.18. ^1^H NMR (CDCl_3_, δ, ppm): 1.29 and 1.46 (both s, each 18 H, tBu), 2.84 (s, 12H, DABCO), 6.71 (d, ^4^J(H,H) = 2.3 Hz, 2 H, arom. C_6_H_2_), 6.96 (d, ^4^J(H,H) = 2.3 Hz, 2 H, arom. C_6_H_2_), 7.39–7.55 (m, 18 H, *o,p*-H, Ph), 7.71–7.83 (m, 12 H, *m*-H, Ph). ^13^C{^1^H} NMR (CDCl_3_, δ, ppm): 29.75, 31.78, 34.44, 34.67, 46.97, 107.81, 112.49, 129.13, 121.04, 133.23, 135.08, 138.12, 139.58, 143.01.

#### 3.3.11. [(4,5-. pip-3,6-DBCat)SbPh_3_]_2_·(DABCO) (**10**)

Orange crystals were isolated from toluene. Yield is 70%. Calcd. for C_78_H_94_N_6_O_4_Sb_2_ (%): C, 65.82; H, 6.61; Sb, 17.16. Found (%): C, 65.74; H, 6.53; Sb, 17.28. IR (nujol, cm^−1^): 1432 m (ν arom. C–C); 1332 m, 1303 w, 1290 w, 1271 w, 1264 w, 1225 m (ν C–O), 1188 m, 1156 w, 1072 m, 1056 s, 1042 s, 1022 w, 998 s, 981 s, 935 m, 854 m, 820 w; 771 s, 732 s, 697 s (δ SbPh_3_); 661 m, 623 w, 594 w, 559 m, 532 m, 521 m, 468 s, 455 s (ν Sb–O). ^1^H NMR (CDCl_3_, δ, ppm): 1.61 (c, 36H, tBu), 2.58–2.70 (m, 8 H, N(CH_2_CH_2_)_2_N), 2.89–3.03 (m, 20 H: 8 H, N(CH_2_CH_2_)_2_N + 12H, DABCO), 7.39–7.53 (m, 18 H, *o,p*-H, Ph), 7.70–7.82 (m, 12 H, *m*-H, Ph). ^13^C{^1^H} NMR (CDCl_3_, δ, ppm): 32.33, 35.46, 46.54, 50.14, 126.47, 128.20, 128.99, 130.90, 135.07, 138.14, 141.19, 143.63.

#### 3.3.12. [(4,5-. Cl_2_-3,6-DBCat)SbPh_3_]_2_·(DABCO) (**11**)

The fine crystalline yellow product was isolated from a toluene–n-hexane (1:1) mixture. Yield is 75%. Calcd. for C_70_H_78_ Cl_4_N_2_O_4_Sb_2_ (%): C, 60.19; H, 5.63; Sb, 17.44. Found (%): C, 60.04; H, 5.55; Sb, 17.38. IR (nujol, cm^−1^): 1434 m (ν arom. C–C); 1395 m, 1318 w, 1302 w, 1253 m (ν C–O), 1209 m, 1167 w, 1074 w, 1058 m, 1032 w, 1022 w, 997 m, 978 m, 927 w, 839 s; 775 s, 730 s, 694 s (δ SbPh_3_); 670 w, 661 w, 618 w, 593 w, 498 m, 466 m, 453 m (ν Sb–O). ^1^H NMR (CDCl_3_, δ, ppm): 1.63 (s, 36 H, tBu), 2.89 (c, 12H, DABCO), 7.40–7.56 (m, 18 H, *o,p*-H, Ph), 7.65–7.77 (m, 12 H, *m*-H, Ph). ^13^C{^1^H} NMR (CDCl_3_, δ, ppm): 32.21, 38.49, 46.53, 123.68, 129.27,129.73, 131.30, 134.74, 137.22, 145.55.

#### 3.3.13. {[(4,5-. Cl_2_-3,6-DBCat)SbPh_3_·H_2_O]·DABCO}_n_ (**12**)

The fine crystalline yellow product was isolated from a toluene–n-hexane (2:1) mixture as a result of a slow solvent evaporation in open atmosphere at ambient temperature. Yield is 67%. ^1^H NMR (CDCl_3_, δ, ppm): 1.62 (s, 36 H, tBu), 2.81 (c, 24H, DABCO), 7.41–7.54 (m, 18 H, *o,p*-H, Ph), 7.65–7.73 (m, 12 H, *m*-H, Ph). ^13^C{^1^H} NMR (CDCl_3_, δ, ppm): 32.19, 38.47, 46.86, 123.62, 129.22,129.68, 131.23, 134.72, 137.37, 145.57.

#### 3.3.14. Tetranuclear complex {Ph_3_Sb(Cat-Spiro-Cat)SbPh_3_∙(Bipy)}_2_ (**13**)

Yellow-orange crystalline powder was isolated by the prolonged crystallization from a toluene solution. Yield is 81%. Calcd. for C_134_H_116_N_4_O_8_Sb_4_ (%): C, 67.13; H, 4.88; Sb, 20.32. Found (%): C, 67.05; H, 4.77; Sb, 20.50. ^1^H NMR (CDCl_3_, δ, ppm): 1.26 and 1.29 (both s, each 12 H, CH_3_), 2.18 (m, 8H, CH_2_), 6.35 and 6.69 (both s, each 4 H, arom. C_6_H_2_), 7.30–7.70 (m, 36 H, *o,p*-H, Ph + 8 H, Bipy), 7.65–7.77 (m, 24 H, *m*-H, Ph), 8.60–8.65 (m, 8 H, Bipy). ^13^C{^1^H} NMR (CDCl_3_, δ, ppm): 30.73, 31.89, 43.13, 57.58, 59.78, 105.01, 107.77, 121.39, 126.42, 129.08, 129.57, 130.87, 131.72, 134.11, 135.11, 138.48, 145.53, 150.46.

## 4. Conclusions

Thus, we have synthesized and investigated the crystal structure and electrochemical behavior of a series of binuclear antimony(V) complexes **1**–**11** of the general type (Cat)Ph_3_Sb-linker-SbPh_3_(Cat), with redox-active catecholate ligands using neutral nitro-gen-containing linkers (pyrazine, 4,4′-dipyridyl, bis-(pyridine-4-yl)-disulfide, diazobi-cyclo[2,2,2]octane). The reaction proceeds easily with high preparative yields but requires dry media, otherwise the formation of polymeric species like {[(4,5-Cl_2_-3,6-DBCat)SbPh_3_∙H_2_O]∙DABCO}_n_ (**12**) may be a side result. Moreover, a rare tetranuclear triphenylantimony(V) spiro-bis-catecholate {Ph_3_Sb(Cat-Spiro-Cat)SbPh_3_∙(Bipy)}_2_ (**13**) was successfully prepared from binuclear complex Ph_3_Sb(Cat-Spiro-Cat)SbPh_3_. Complexes demonstrate different types of relative spatial positions of mononuclear moieties depending on the nature of both Cat and linker ligands. The nature of chemical bonds, charges distribution, and the energy of Sb...N interaction were investigated in the example of complex **5**. The electrochemical behavior of the complexes depends on the coordinated N-donor ligand.

Complexes of this type with redox-active ligands represent a useful Sinton for the design of heterospin derivatives, based on redox-active centers of different natures (e.g., Cat, SQ ligands, and N-donor linkers, including paramagnetic linkers, etc.).

## Figures and Tables

**Table 1:** Basic topological characteristics of the electron density in **5_ED_**.

Bond	*ν*(r), a.e.	*ρ*(r), a.e.	∇^2^*ρ*(r), a.e.	*h*_e_(r), a.e.
Sb(1)–O(1)	−0.178	0.112	0.332	−0.047
Sb(1)–O(2)	−0.144	0.092	0.422	−0.019
Sb(1)–N(1)	−0.024	0.030	0.083	−0.002
Sb(1)–C(15)	−0.132	0.098	0.141	−0.048
Sb(1)–C(21)	−0.156	0.111	0.105	−0.065
Sb(1)–C(27)	−0.188	0.126	0.064	−0.086
O(1)–C(1)	−0.711	0.300	−0.741	−0.448
O(2)–C(2)	−0.726	0.304	−0.796	−0.462
C(1)–C(2)	−0.772	0.315	−0.781	−0.484

**Table 2:** The electrochemical potentials for **1–7**, **9**, and **13** in the accordance with CV (GC, CH_2_Cl_2_, V = 0.2 V∙s^−1^, 0.1 M Bu_4_NClO_4_, C = 3 × 10^−3^ M, Ar, Ag/AgCl/KCl(sat.)).

Complex	E^1^_pa_, V	E^2^_pa_, V	E^3^_pa_, V
**1** [(3,6-DBCat)SbPh_3_]_2_·(Pyr)	0.93	-	-
**2** [(3,6-DBCat)SbPh_3_]_2_·(PySSPy)	0.97	-	-
**3** [(3,6-DBCat)SbPh_3_]_2_·(Bipy)	0.96	-	-
**4** [(3,6-DBCat)SbPh_3_]_2_·(DABCO)	0.45 *	0.76	0.91
**5** [(3,5-DBCat)SbPh_3_]_2_·(Bipy)	0.95	-	-
**6** [(4,5-pip-3,6-DBCat)SbPh_3_]_2_·(Bipy)	0.73	1.15	1.46
**7** [(4,5-Cl_2_-3,6-DBCat)SbPh_3_]_2_·(Bipy)	1.05	-	-
**9** [(3,5-DBCat)SbPh_3_]_2_·(DABCO)	0.47 *	0.80	0.88
**13**{Ph_3_Sb(Cat-Spiro-Cat)SbPh_3_∙(Bipy)}_2_	0.83	1.05	-

E^1^_pa_—the peak potential for the first oxidation process; E^2^_p__a_—the peak potential for the second oxidation process; E^3^_pa_—the peak potential for the third oxidation process. *—the half-wave potential value.

## Data Availability

The data presented in this study are available in this article.

## Supplementary Materials

The following supporting information can be downloaded at: https://www.mdpi.com/article/10.3390/molecules27196484/s1. Figures S1–S26: ^1^H and ^13^C{^1^H} NMR spectra for complexes **1**–**13**; Figures S27–S34: The different views on the molecules of **1**, **3**, **4**, **5**, **8**, **10**, **12**, and **13** in crystals; Figure S35: F_o_/F_c_ vs resolution; Figure S36: Normal probability; Figure S37: Fractal dimension vs residual density; Figure S38: The direction of the lone electron pair of the nitrogen atom in Bipy in **5_ED_**; Table S1: The details of X-ray experiment and structure refinement; Table S2: The selected bond lengths in complexes in the accordance with the bonds scheme; Table S3: The selected structural parameters for complexes; The data on Multipole refinement of **5_ED_** (Citations: [89,90,91,92,93]); CCDC 2174897 (**1**), 2174898 (**3**), 2174899 (**4**∙n-hexane), 2174900 (**5**; low resolution-IAM), 2174901 (**5**; high resolution-IAM), 2174902 (**5**; high resolution multipole refinement), 2174903 (**8**∙toluene), 2174904 (**10∙**3.5toluene), 2174905 (**12∙**0.25toluene) and 2174906 (**13**∙6toluene) contain the supplementary crystallographic data for this paper. These data can be obtained free of charge from The Cambridge Crystallographic Data Centre via www.ccdc.cam.ac.uk/data_request/cif (accessed on 19 September 2022).

## Sample Availability

Samples of the compounds **1**–**13** are available from the authors.
