# Supplementary material for: Binuclear Triphenylantimony(V) Catecholates through N-Donor Linkers: Structural Features and Redox Properties
Source: Molecules. 2022 Oct 1;27(19):6484. doi: 10.3390/molecules27196484 (PMC9573088; doi:10.3390/molecules27196484)
Supplement: Supplementary file 1 [file molecules-27-06484-s001.zip › molecules-1952312-supplementary.pdf]

Supplementary information

Binuclear triphenylantimony(V) catecholates through N-donor linkers: structural features and redox properties

Andrey I. Poddel'sky, Ivan V. Smolyaninov, Aleksandra I. Shataeva,
Evgenii V. Baranov, Georgy K. Fukin

Content:

Figure S1. The ^{1}H NMR spectrum of **1** ($CDCl_3$) S3
Figure S2. The $^{13}C\{^{1}H\}$ NMR spectrum of **1** ($CDCl_3$) S3
Figure S3. The ^{1}H NMR spectrum of **2** ($CDCl_3$) S4
Figure S4. The $^{13}C\{^{1}H\}$ NMR spectrum of **2** ($CDCl_3$) S4
Figure S5. The ^{1}H NMR spectrum of **3** ($CDCl_3$) S5
Figure S6. The $^{13}C\{^{1}H\}$ NMR spectrum of **3** ($CDCl_3$) S5
Figure S7. The ^{1}H NMR spectrum of **4** ($CDCl_3$) S6
Figure S8. The $^{13}C\{^{1}H\}$ NMR spectrum of **4** ($CDCl_3$) S6
Figure S9. The ^{1}H NMR spectrum of **5** ($CDCl_3$) S7
Figure S10. The $^{13}C\{^{1}H\}$ NMR spectrum of **5** ($CDCl_3$) S7
Figure S11. The ^{1}H NMR spectrum of **6** ($CDCl_3$) S8
Figure S12. The $^{13}C\{^{1}H\}$ NMR spectrum of **6** ($CDCl_3$) S8
Figure S13. The ^{1}H NMR spectrum of **7** ($CDCl_3$) S9
Figure S14. The $^{13}C\{^{1}H\}$ NMR spectrum of **7** ($CDCl_3$) S9
Figure S15. The ^{1}H NMR spectrum of **8** ($CDCl_3$) S10
Figure S16. The $^{13}C\{^{1}H\}$ NMR spectrum of **8** ($CDCl_3$) S10
Figure S17. The ^{1}H NMR spectrum of **9** ($CDCl_3$) S11
Figure S18. The $^{13}C\{^{1}H\}$ NMR spectrum of **9** ($CDCl_3$) S11
Figure S19. The ^{1}H NMR spectrum of **10** ($CDCl_3$) S12
Figure S20. The $^{13}C\{^{1}H\}$ NMR spectrum of **10** ($CDCl_3$) S12
Figure S21. The ^{1}H NMR spectrum of **11** ($CDCl_3$) S13
Figure S22. The $^{13}C\{^{1}H\}$ NMR spectrum of **11** ($CDCl_3$) S13
Figure S23. The ^{1}H NMR spectrum of **12** ($CDCl_3$) S14
Figure S24. The $^{13}C\{^{1}H\}$ NMR spectrum of **12** ($CDCl_3$) S14
Figure S25. The ^{1}H NMR spectrum of **13** ($CDCl_3$) S15
Figure S26. The $^{13}C\{^{1}H\}$ NMR spectrum of **13** ($CDCl_3$) S15

Table S1. The details of X-ray experiment and structure refinement . . . S16
Table S2. The selected bond lengths in complexes in the accordance with the bonds scheme. S17

Figure S27. The different views on the molecule of **1** in crystal. S18
Figure S28. The different views on the molecule of **3** in crystal. S18
Figure S29. The different views on the molecule of **4** in crystal. S19
Figure S30. The different views on the molecule of **5** in crystal. S19
Figure S31. The different views on the molecule of **8** in crystal. S20
Figure S32. The different views on the molecule of **10** in crystal. . . . S20
Figure S33. The different views on the molecule of **12** in crystal. . . . S21
Figure S34. The different views on the molecule of **13** in crystal. . . . S21

Table S3. The selected structural parameters for complexes. S22

Multipole refinement of $\mathbf{5_{ED}}$ S23
Figure S35. Fo/Fc vs resolution S23
Figure S36. Normal probability S24
Figure S37. Fractal dimension vs residual density S24
Figure S38. The direction of the lone electron pair of the nitrogen atom in Bipy in $\mathbf{5_{ED}}$. . S25
References S25

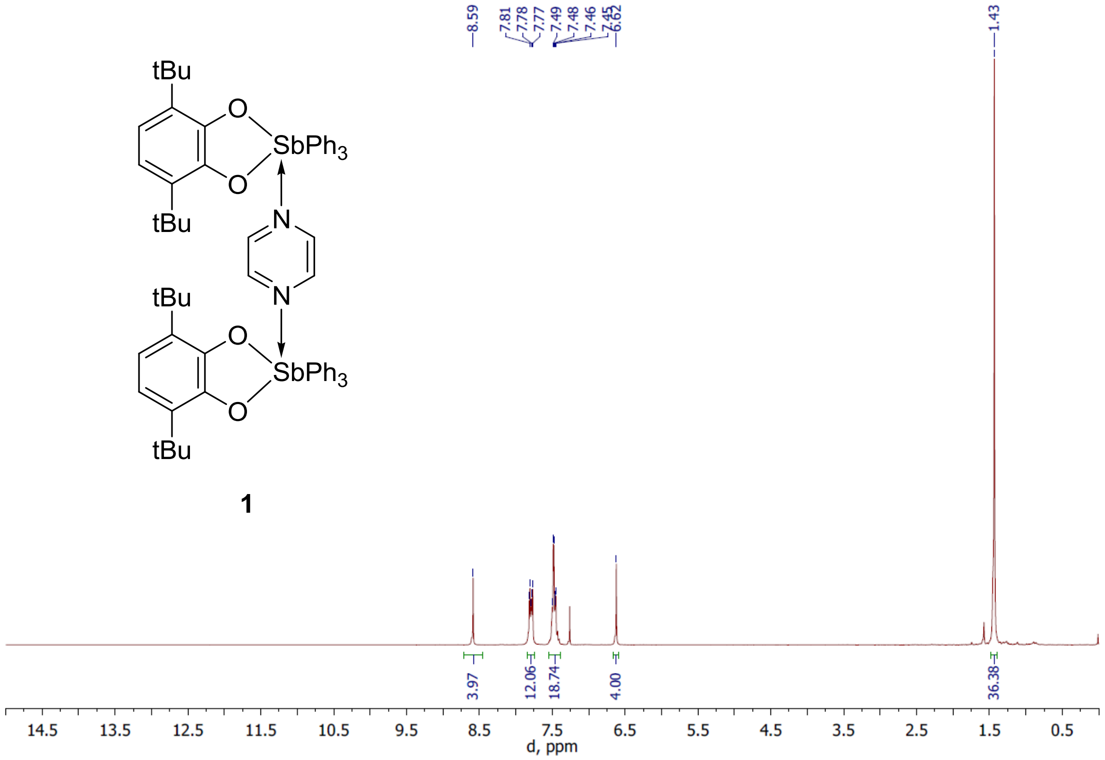

Figure S1. The ^{1}H NMR spectrum of **1** (200 MHz, $CDCl_3$).

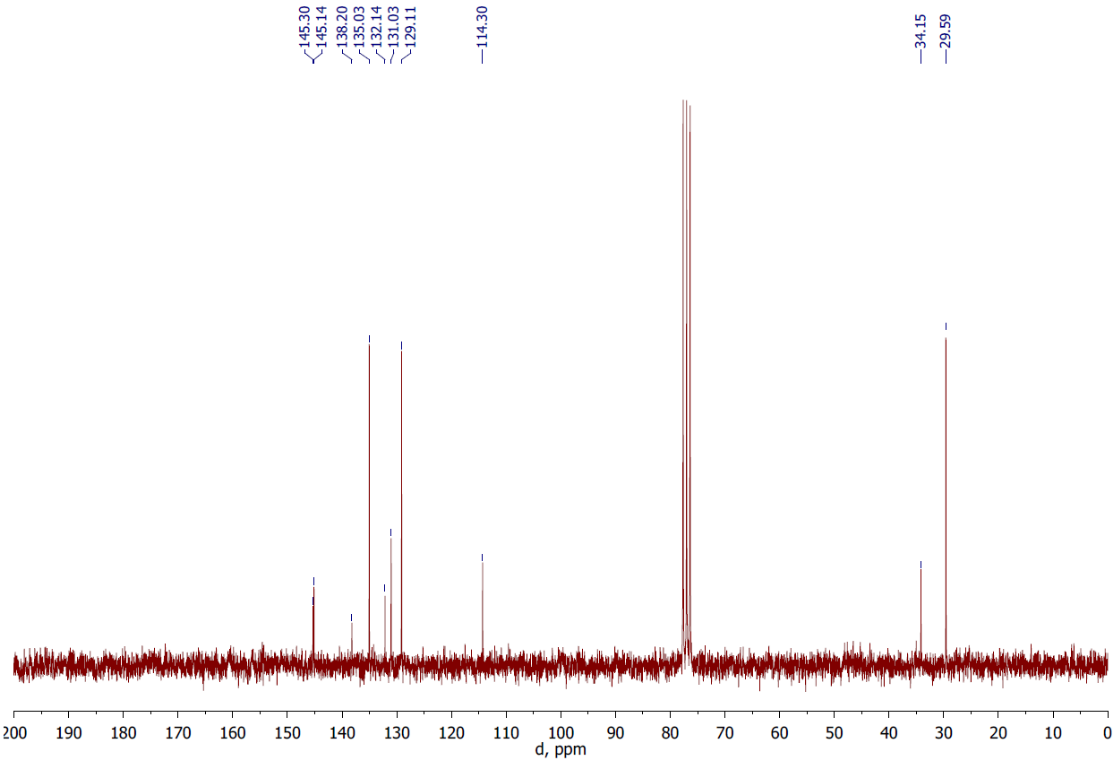

Figure S2. The $^{13}C\{^{1}H\}$ NMR spectrum of **1** (50 MHz, $CDCl_3$).

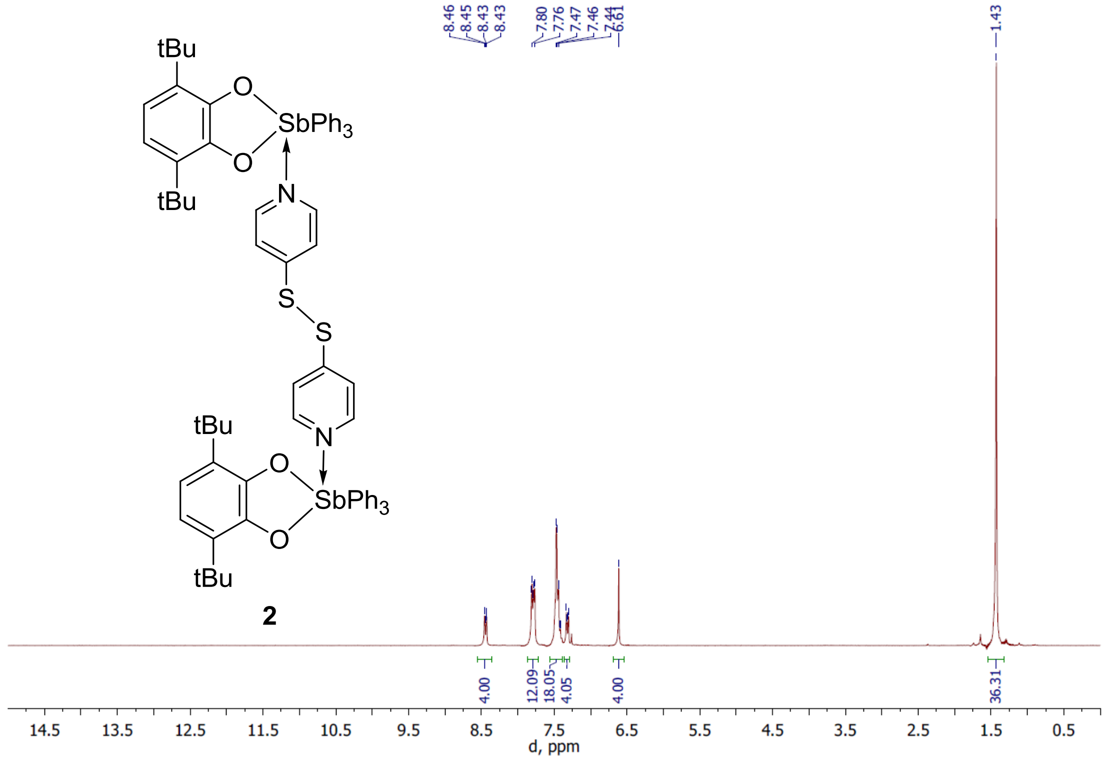

Figure S3. The ^{1}H NMR spectrum of **2** (200 MHz, $CDCl_3$).

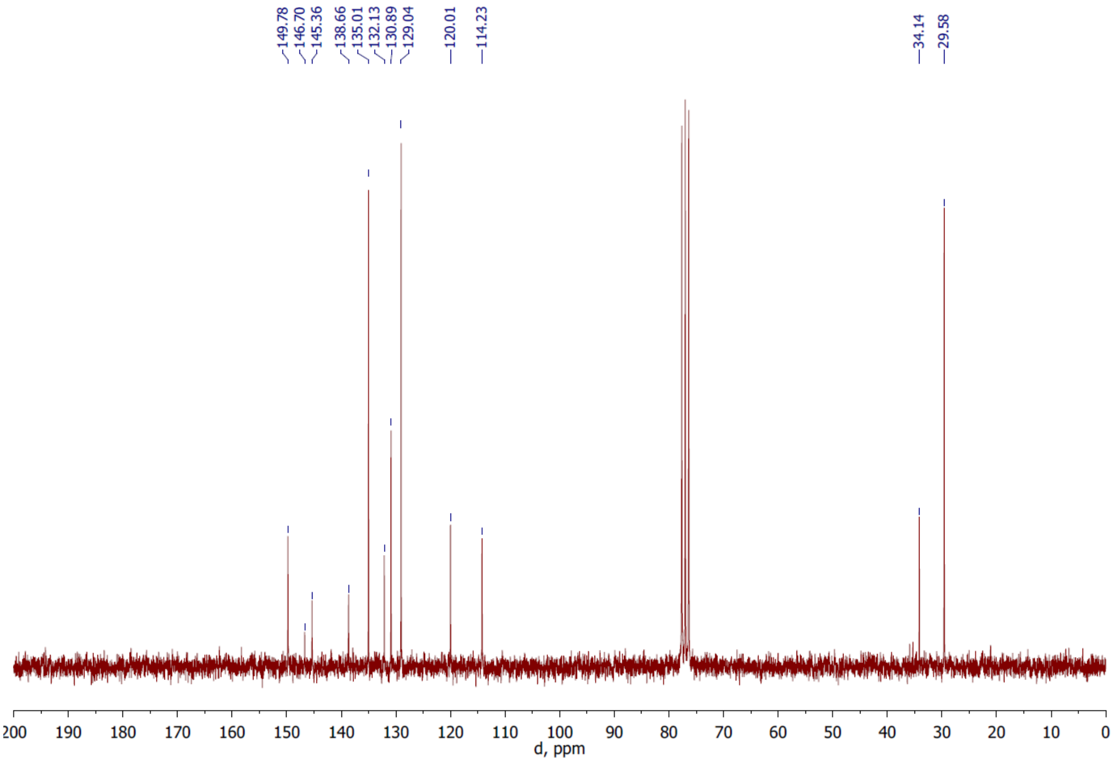

Figure S4. The $^{13}C\{^{1}H\}$ NMR spectrum of **2** (50 MHz, $CDCl_3$).

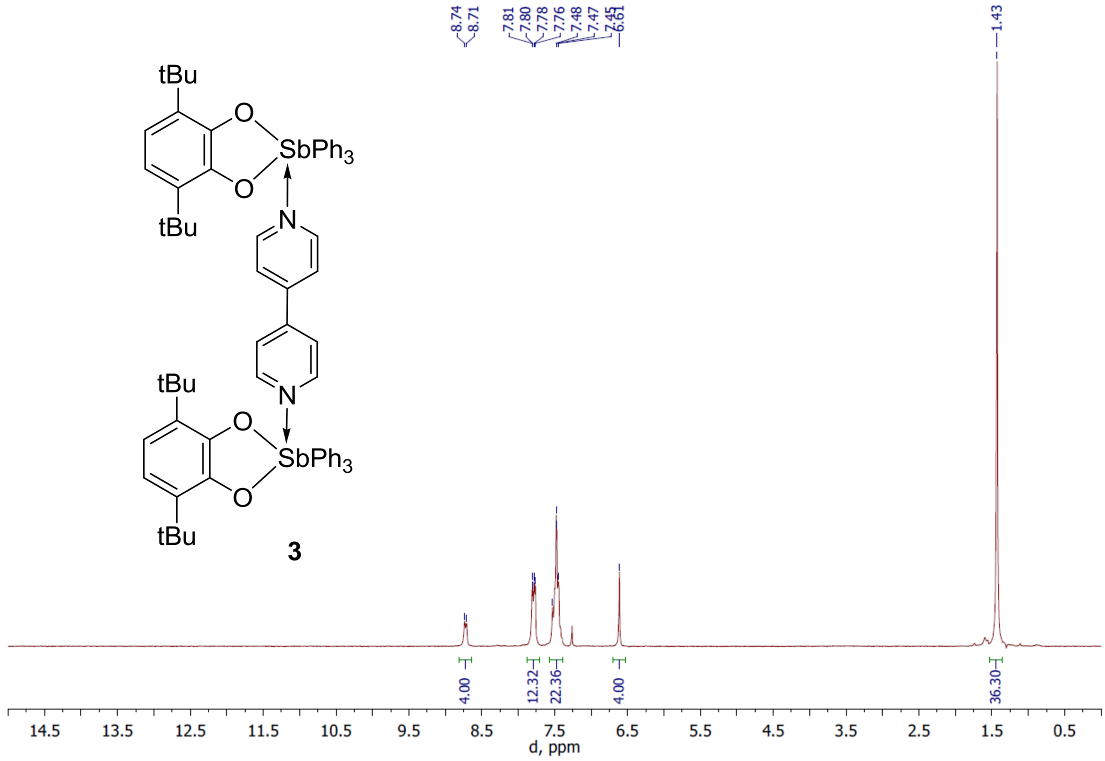

Figure S5. The ^{1}H NMR spectrum of **3** (200 MHz, $CDCl_3$).

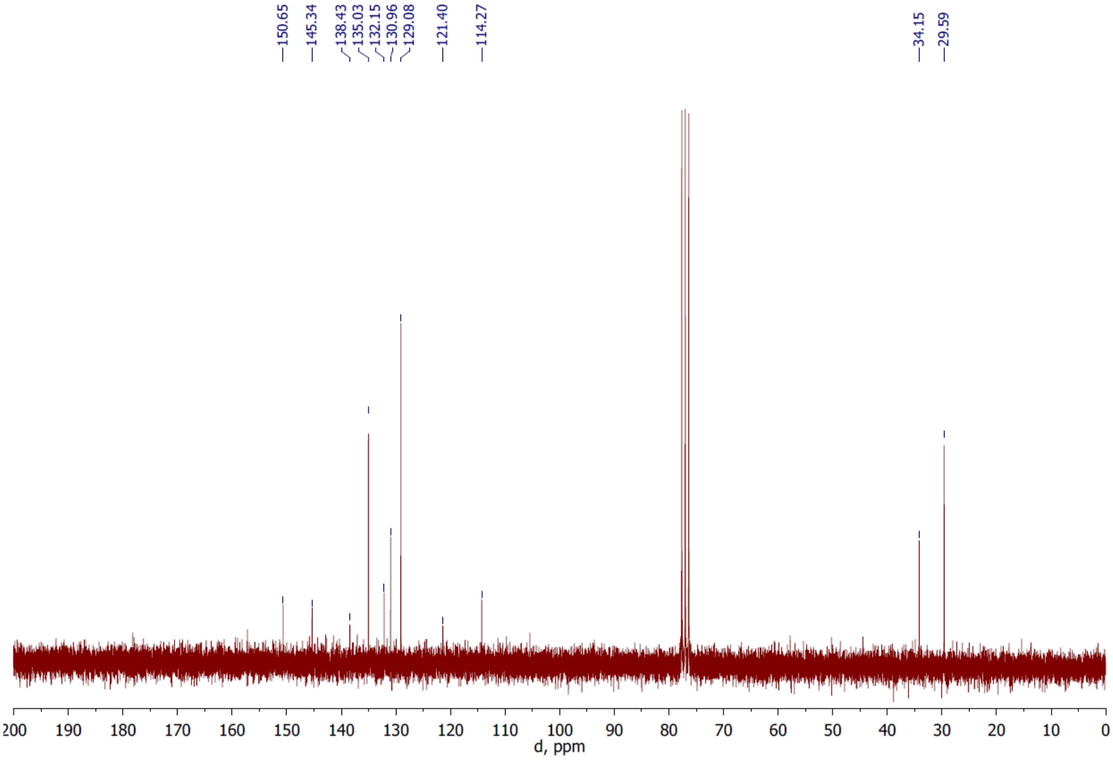

Figure S6. The $^{13}C\{^{1}H\}$ NMR spectrum of **3** (50 MHz, $CDCl_3$) .

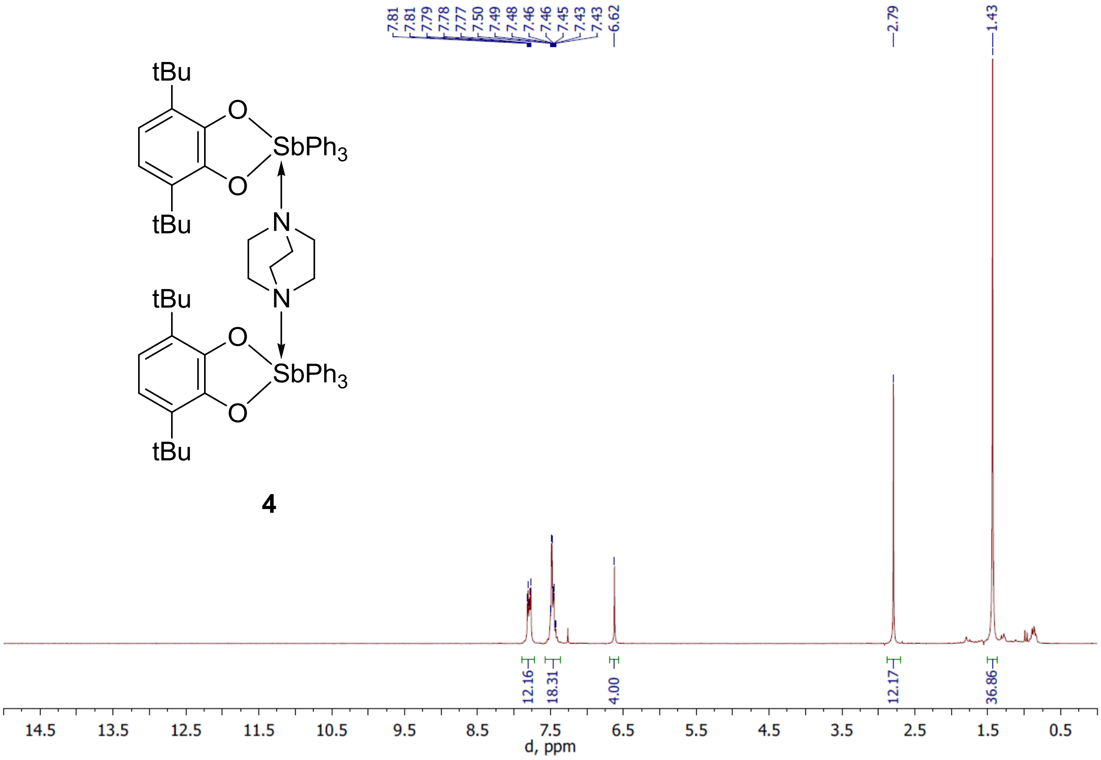

Figure S7. The ^{1}H NMR spectrum of **4** (200 MHz, $CDCl_3$).

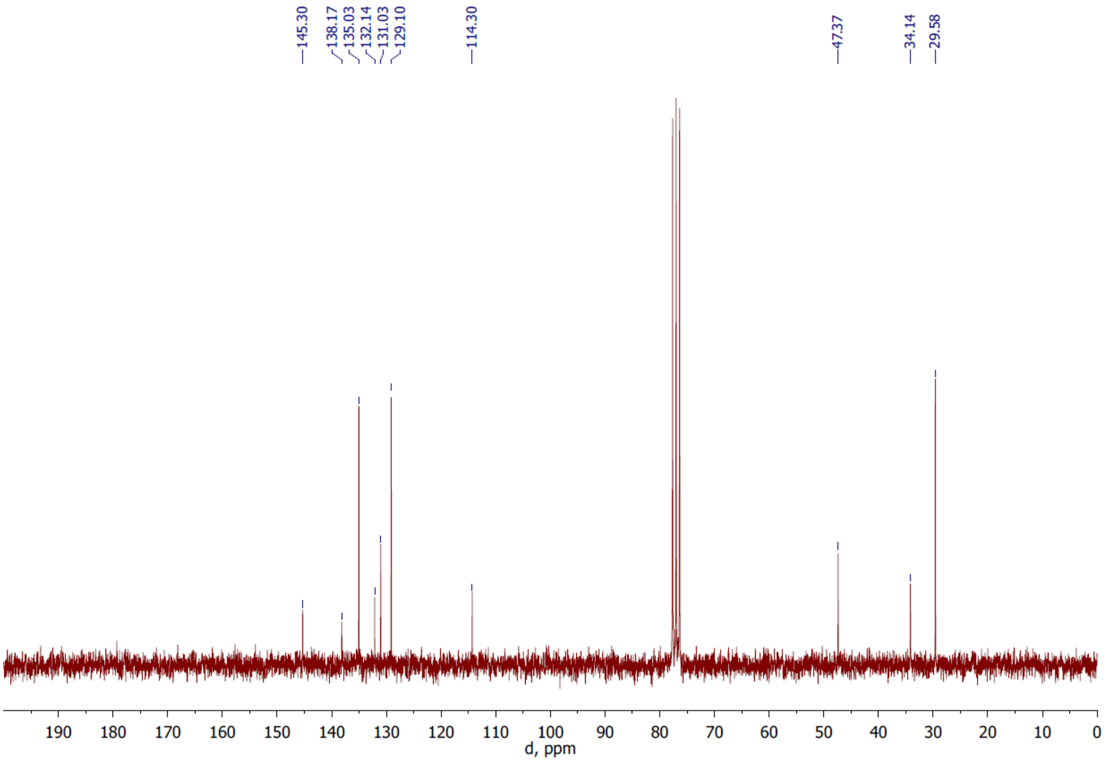

Figure S8. The $^{13}C\{^{1}H\}$ NMR spectrum of **4** (50 MHz, $CDCl_3$).

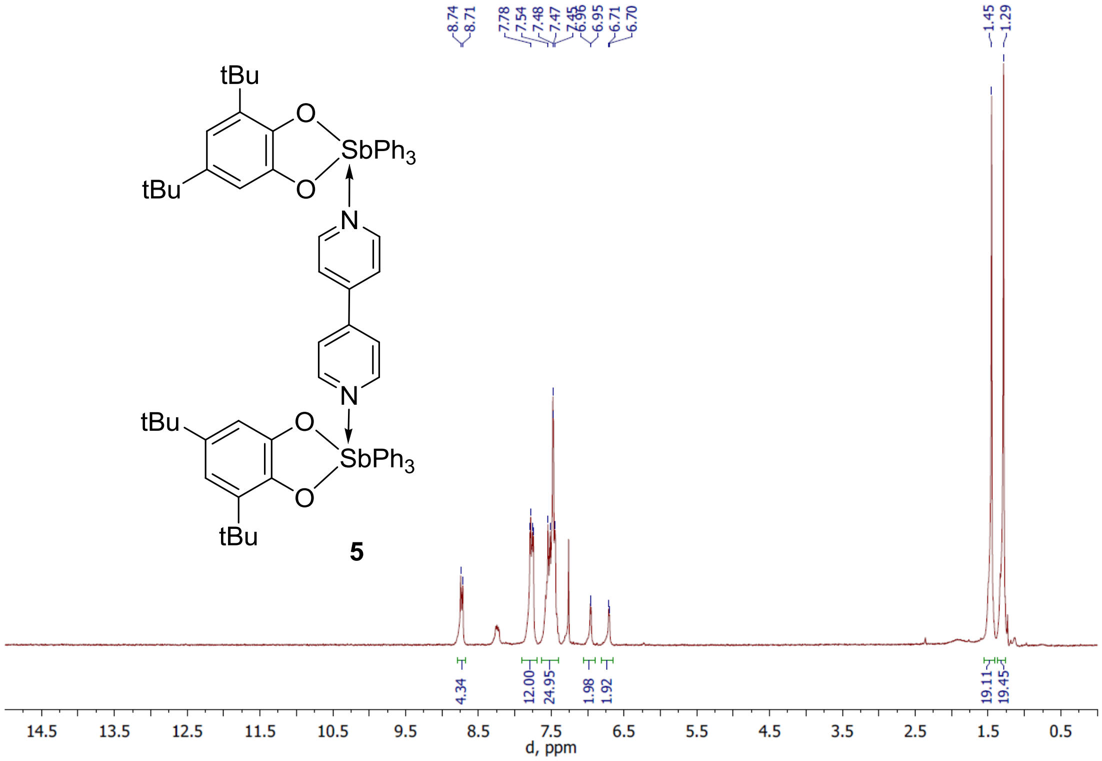

Figure S9. The ^{1}H NMR spectrum of **5** (200 MHz, $CDCl_3$).

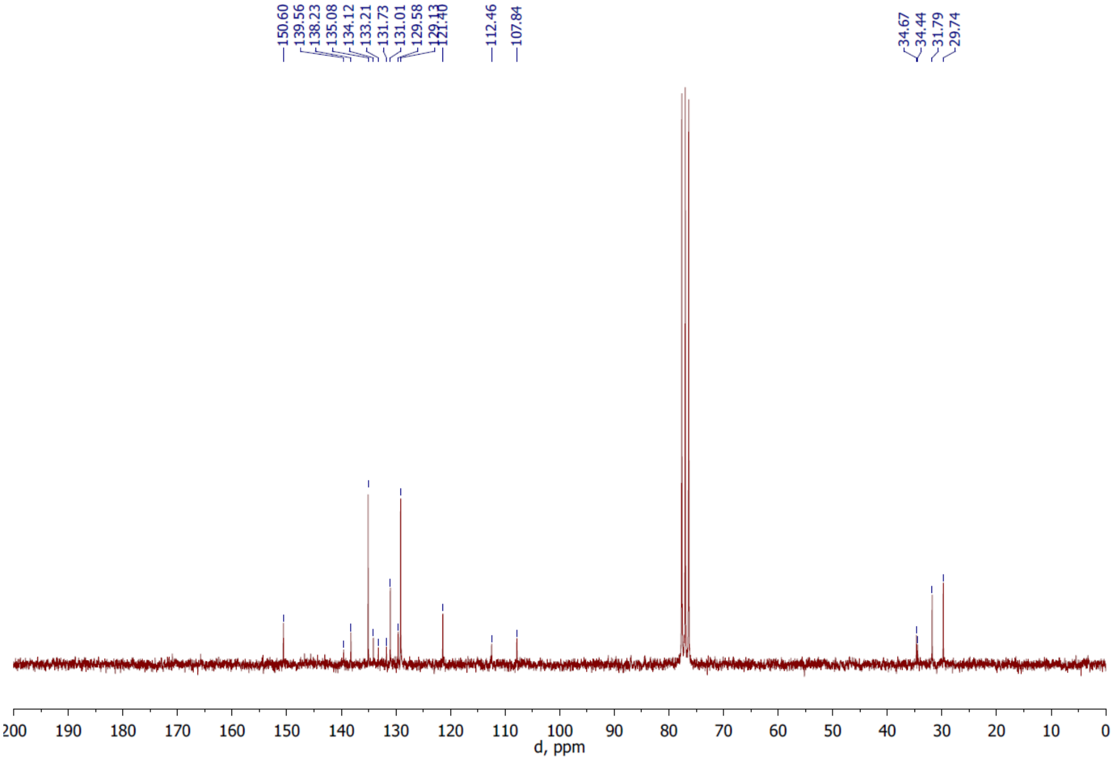

Figure S10. The $^{13}C\{^{1}H\}$ NMR spectrum of **5** (50 MHz, $CDCl_3$).

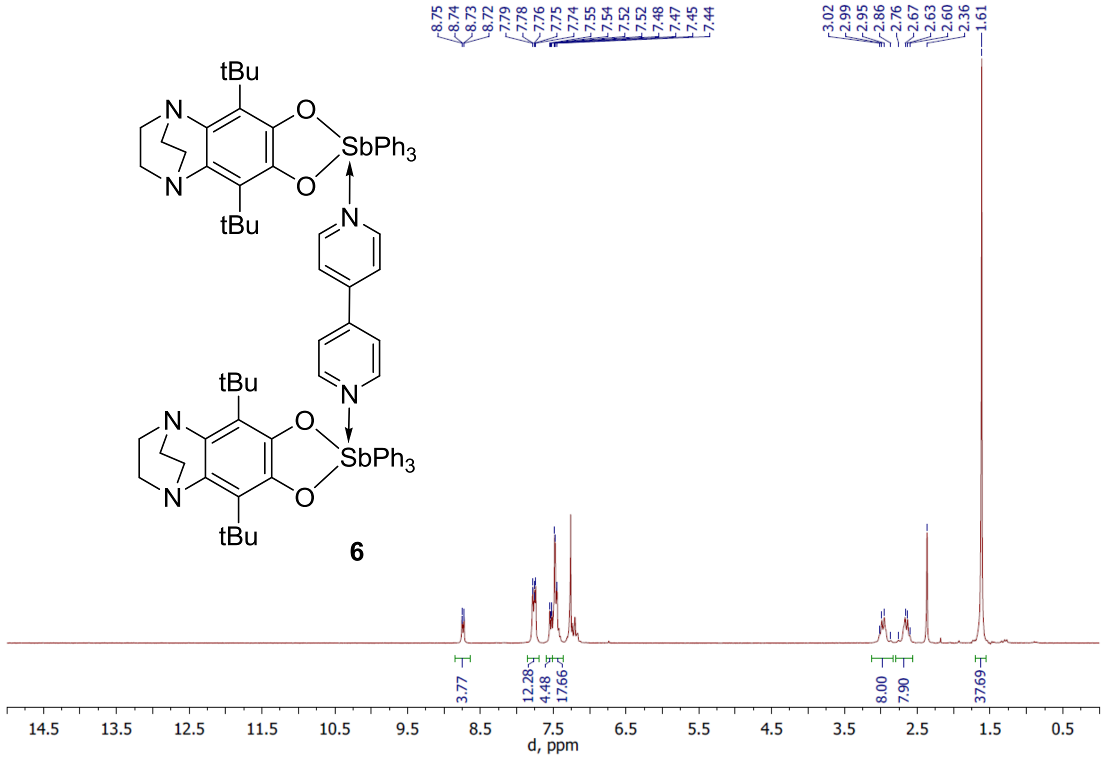

Figure S11. The ^{1}H NMR spectrum of **6** (200 MHz, $CDCl_3$). The peaks of toluene admixture (s. 2.36, m. 7.1-7.3 ppm) are also observed.

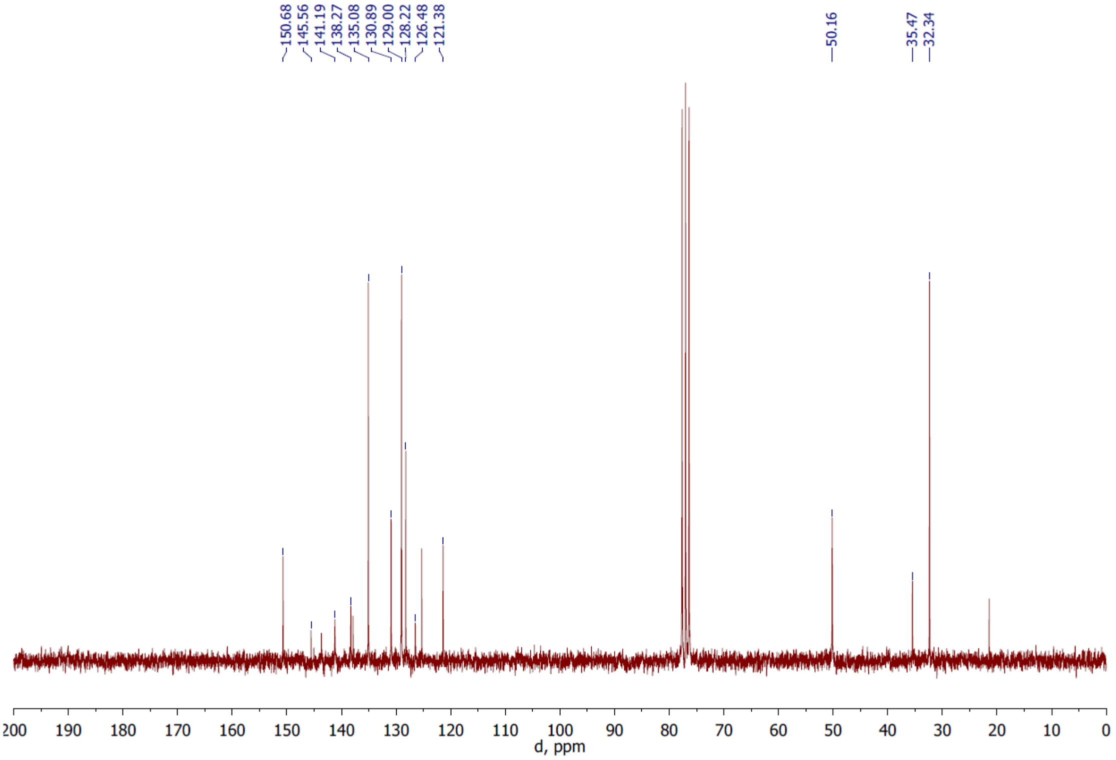

Figure S12. The $^{13}C\{^{1}H\}$ NMR spectrum of **6** (50 MHz, $CDCl_3$). The peaks of toluene admixture (21.46, 125.33, 128.26, 129.07, 137.89 ppm) are also observed.

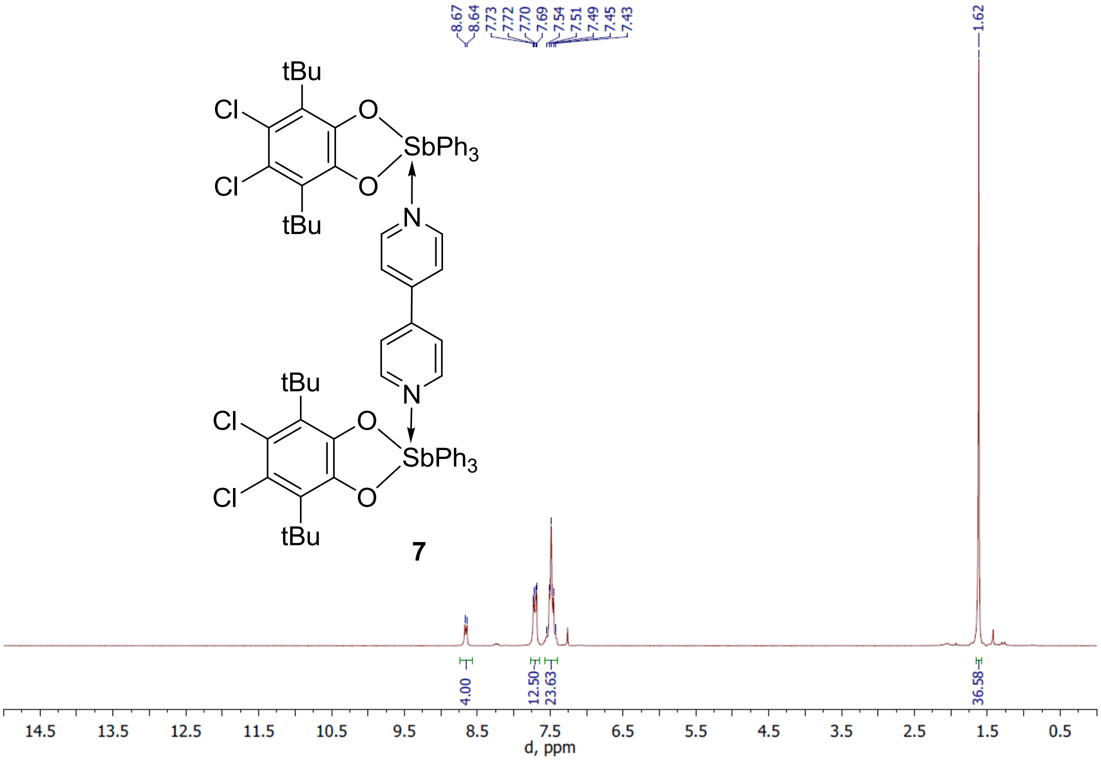

Figure S13. The ^{1}H NMR spectrum of **7** (200 MHz, $CDCl_3$).

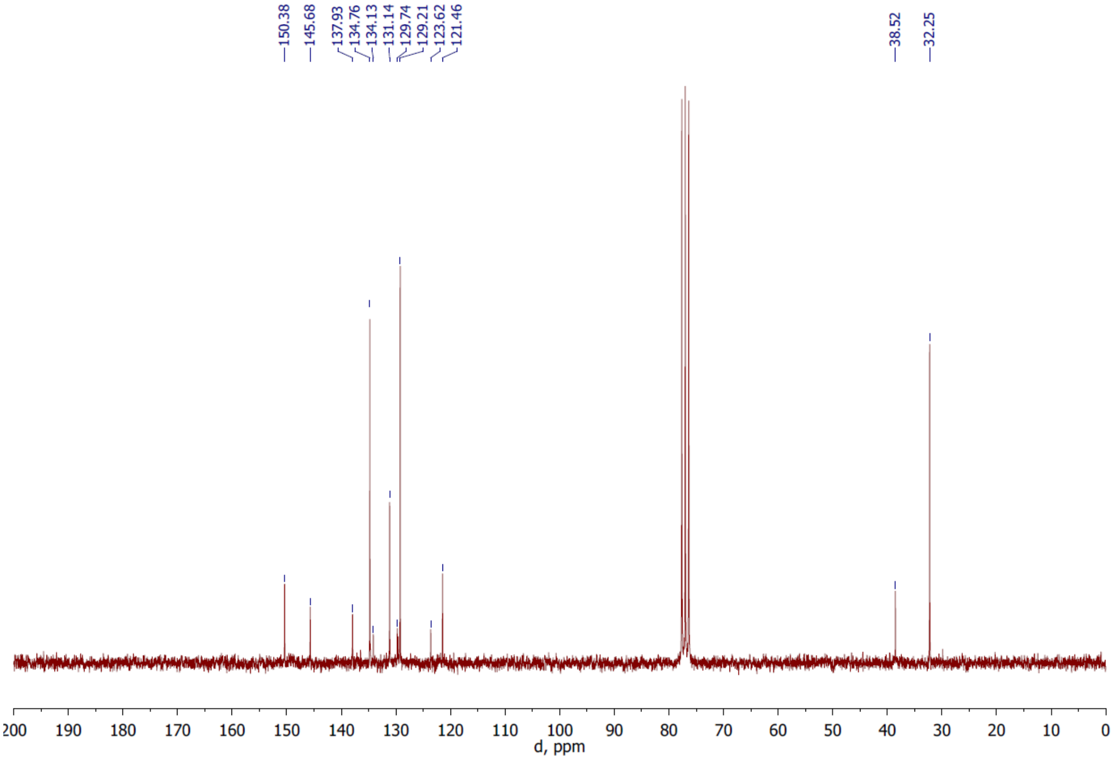

Figure S14. The ^{13}C{^{1}H} NMR spectrum of **7** (50 MHz, $CDCl_3$).

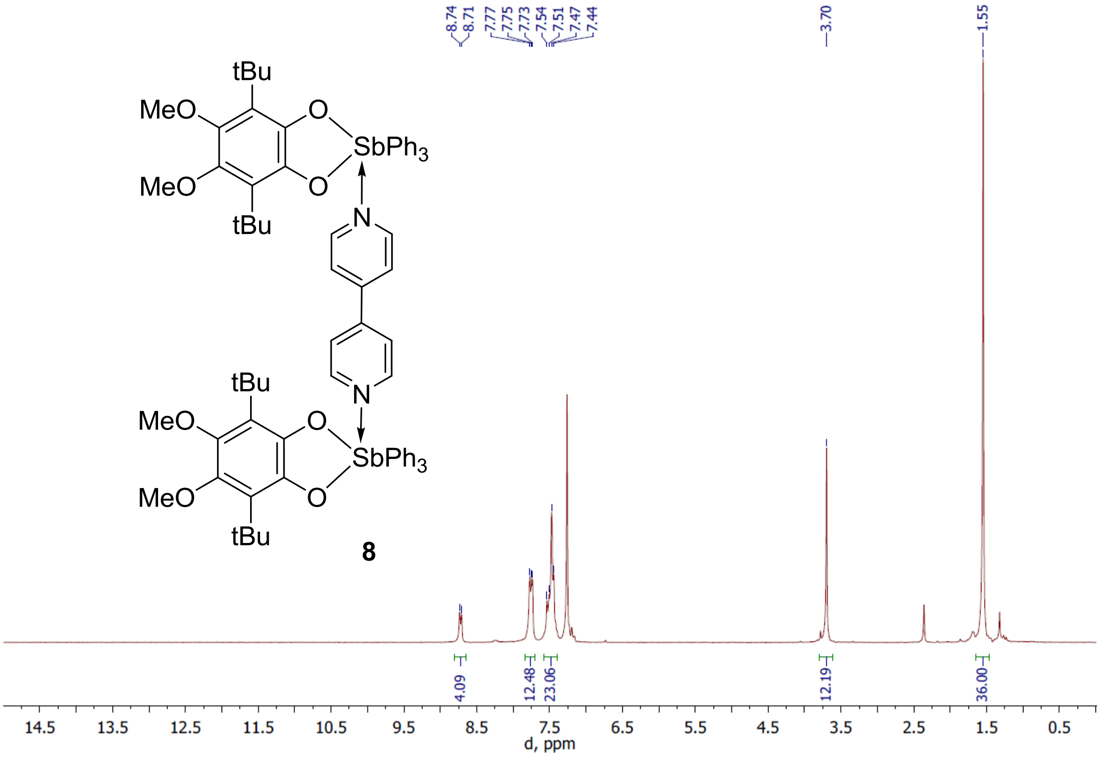

Figure S15. The ^{1}H NMR spectrum of **8** (200 MHz, $CDCl_3$).

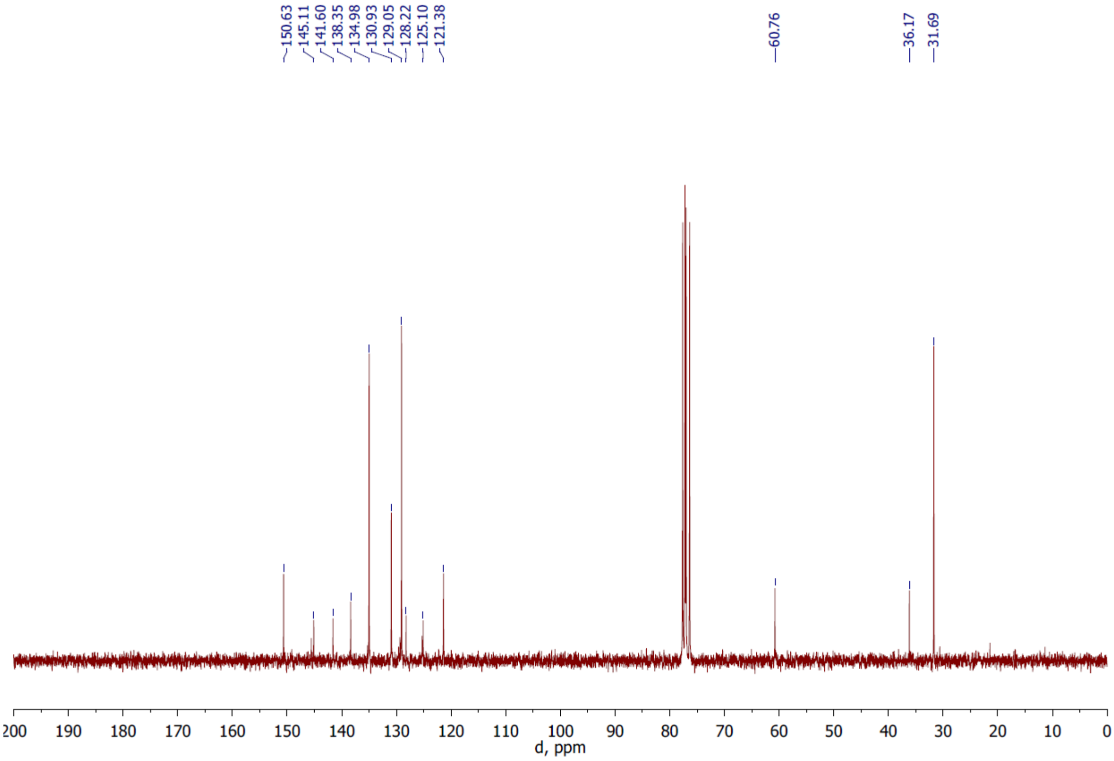

Figure S16. The ^{13}C{^{1}H} NMR spectrum of **8** (50 MHz, $CDCl_3$).

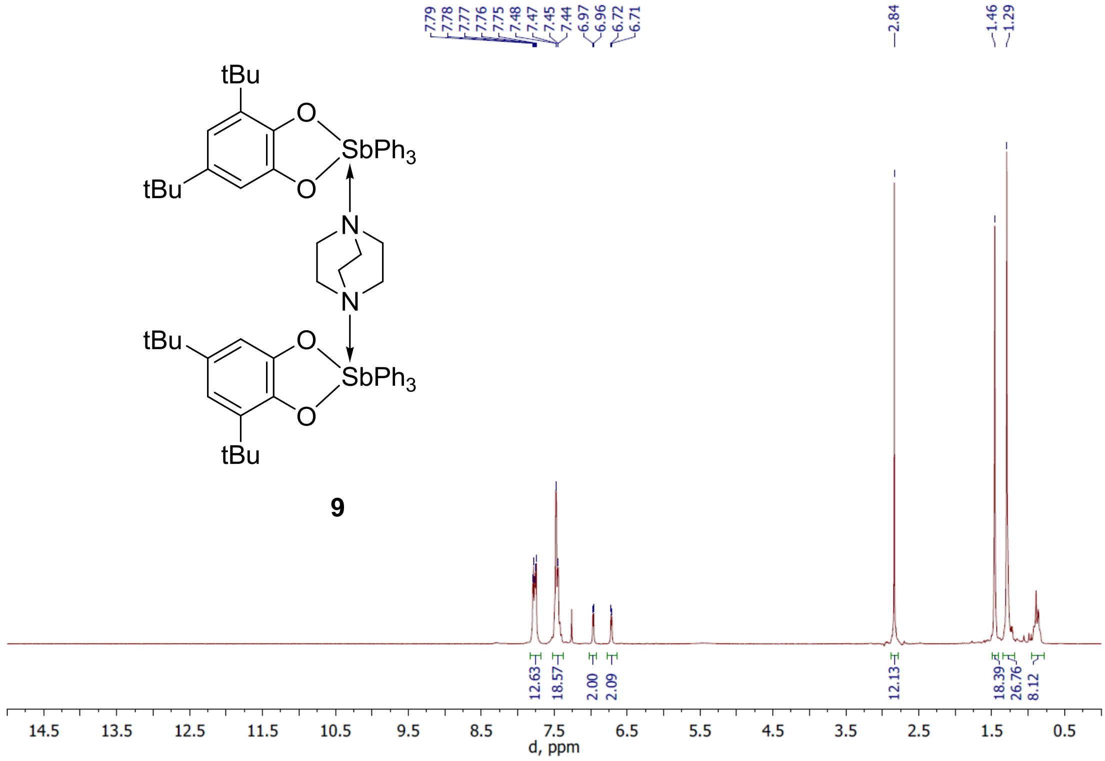

Figure S17. The ^{1}H NMR spectrum of **9** (200 MHz, $CDCl_3$). The peaks of n-hexane admixture (t. 0.88, m. 1.26 ppm) are also observed.

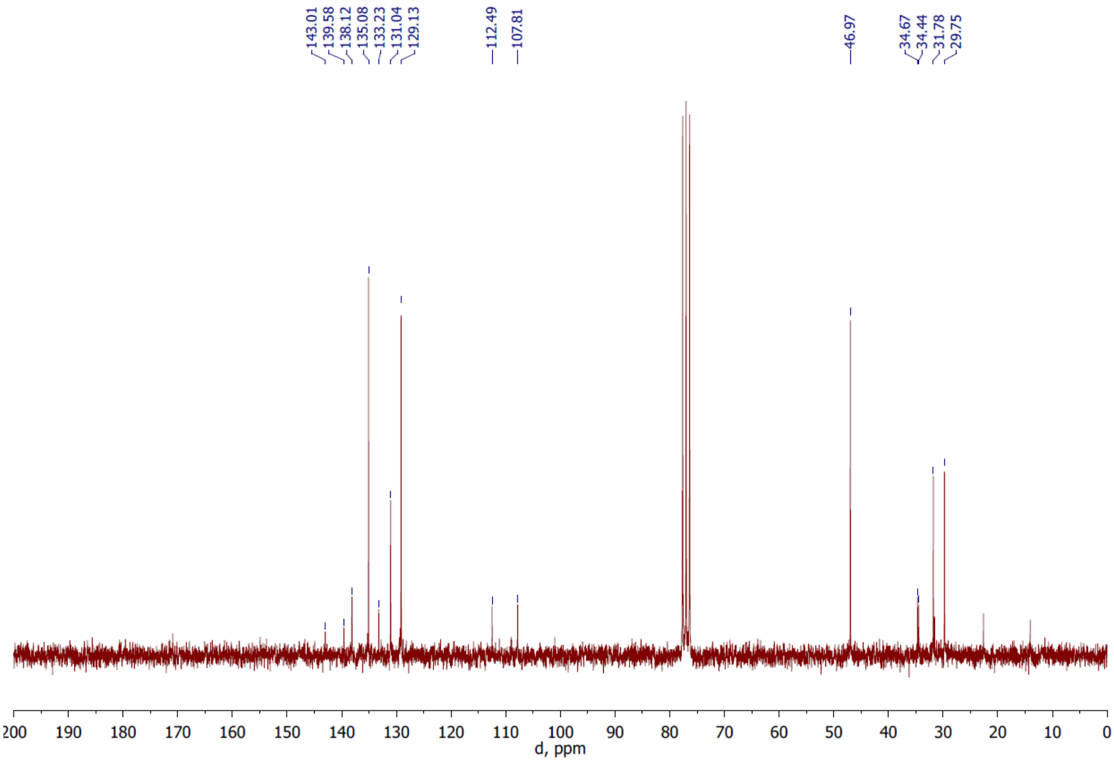

Figure S18. The ^{13}C{^{1}H} NMR spectrum of **9** (50 MHz, $CDCl_3$). The peaks of n-hexane admixture (14.15, 22.70, and 31.64 ppm) are also observed.

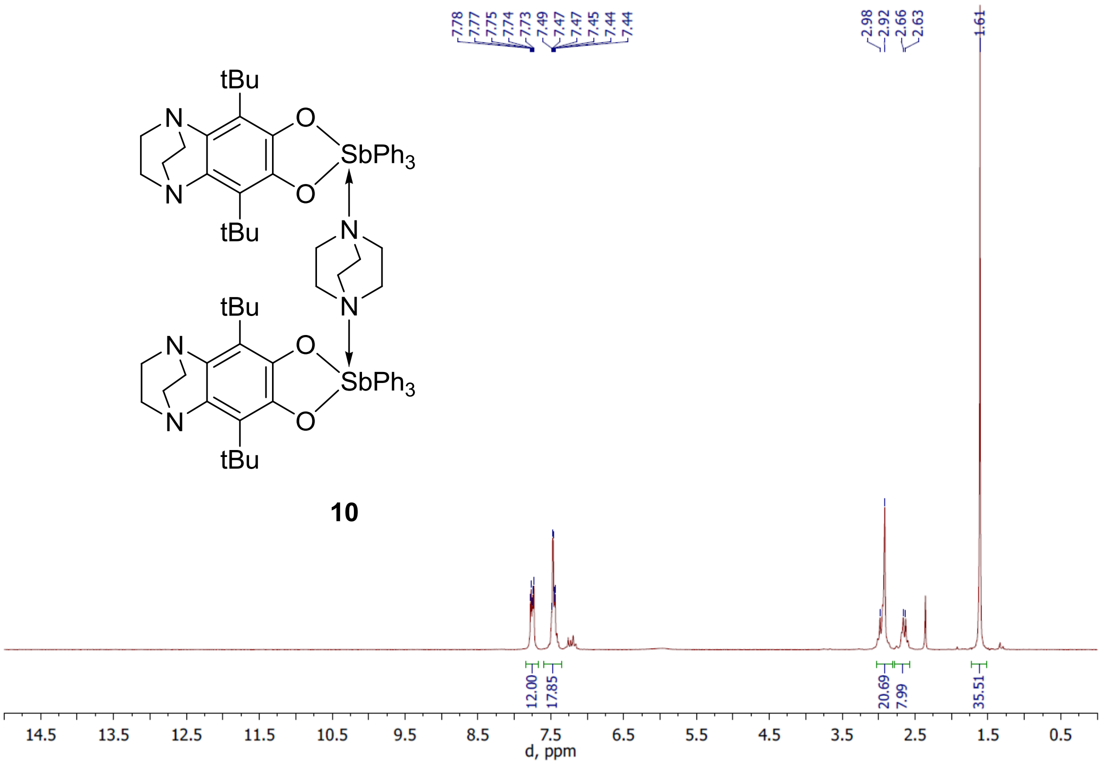

Figure S19. The ^{1}H NMR spectrum of **10** (200 MHz, $CDCl_3$).

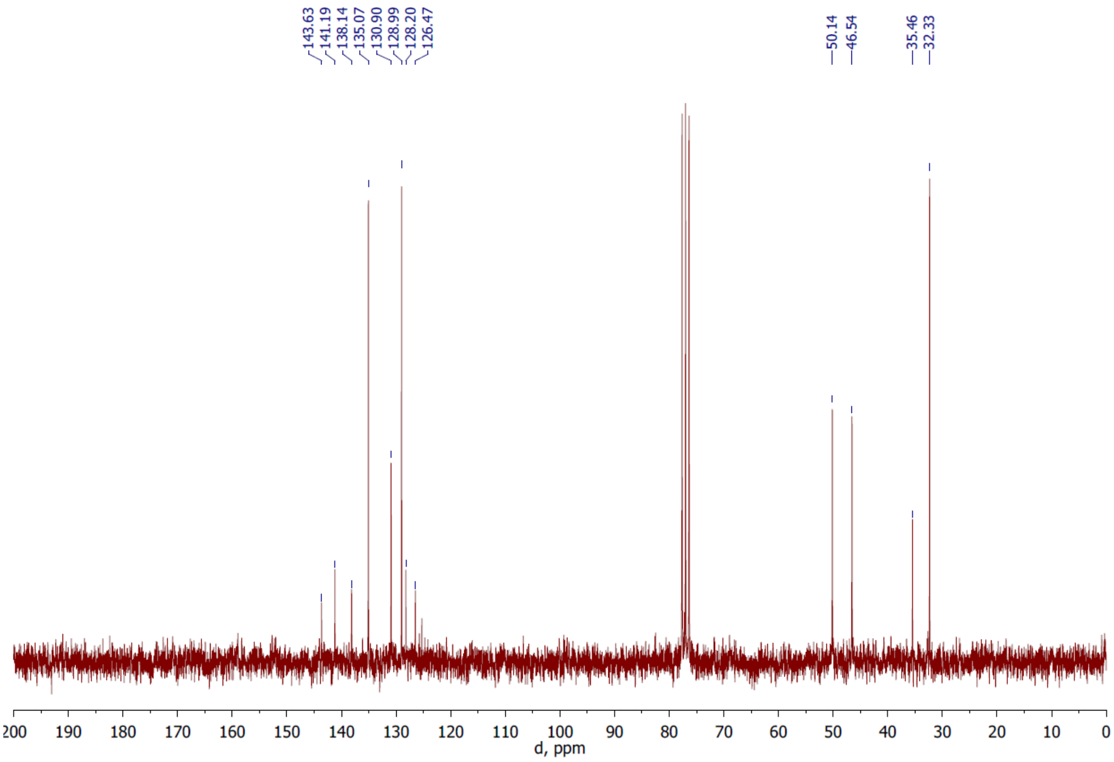

Figure S20. The $^{13}C\{^{1}H\}$ NMR spectrum of **10** (50 MHz, $CDCl_3$).

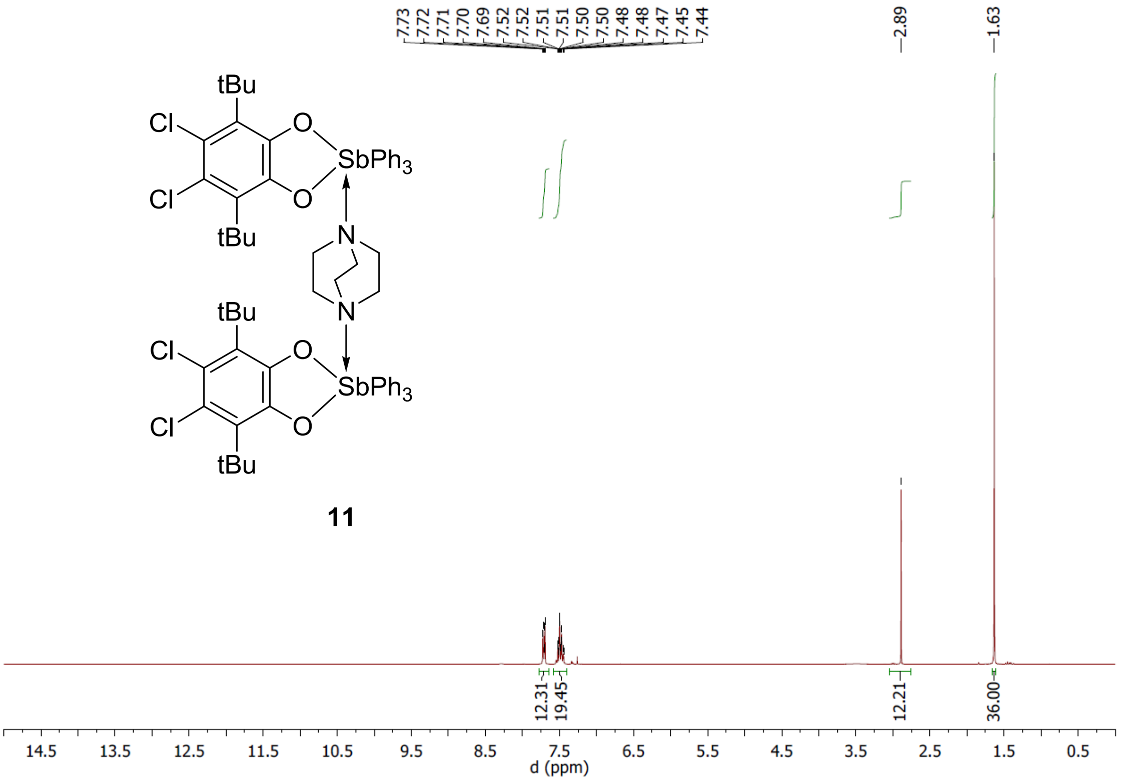

Figure S21. The ^{1}H NMR spectrum of **11** (300 MHz, $CDCl_3$).

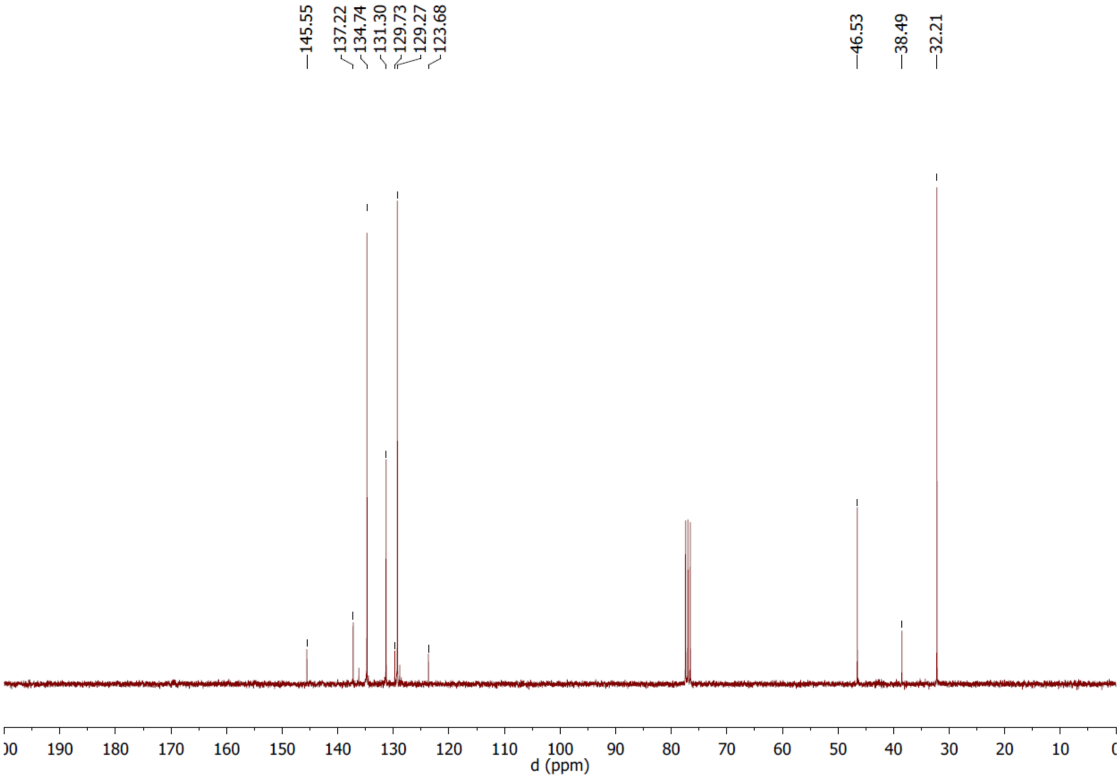

Figure S22. The $^{13}C\{^{1}H\}$ NMR spectrum of **11** (75 MHz, $CDCl_3$).

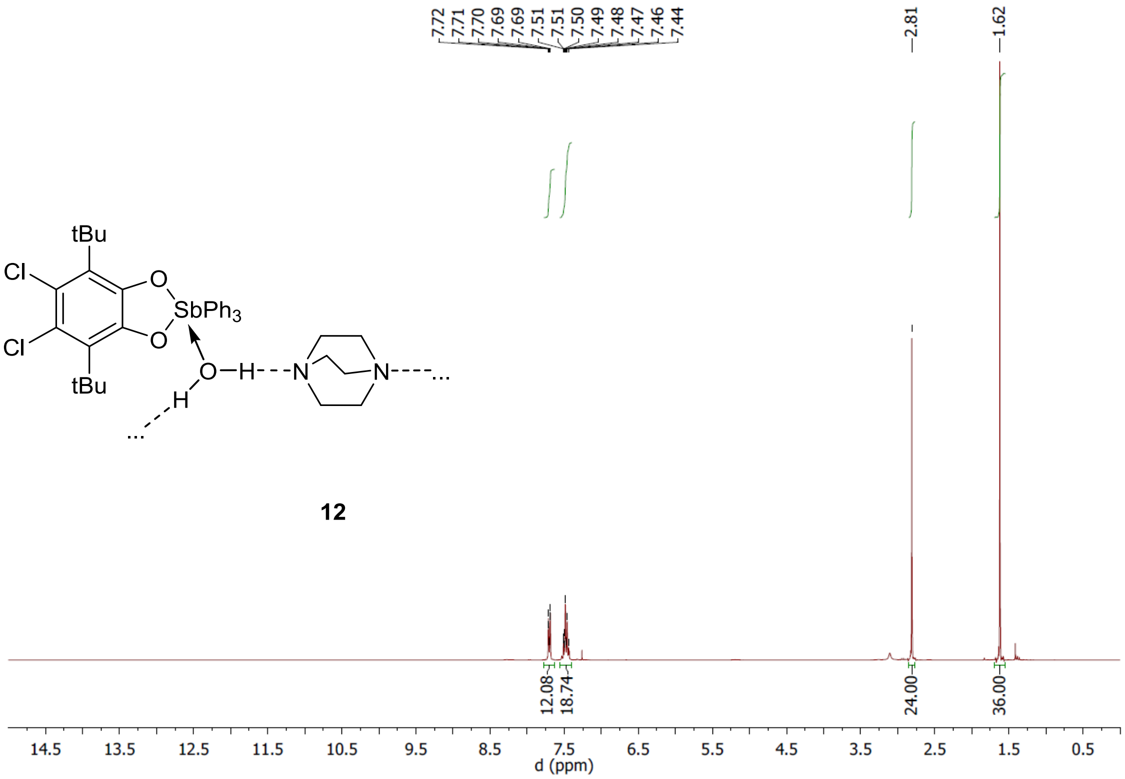

Figure S23. The ^{1}H NMR spectrum of **12** (300 MHz, $CDCl_3$).

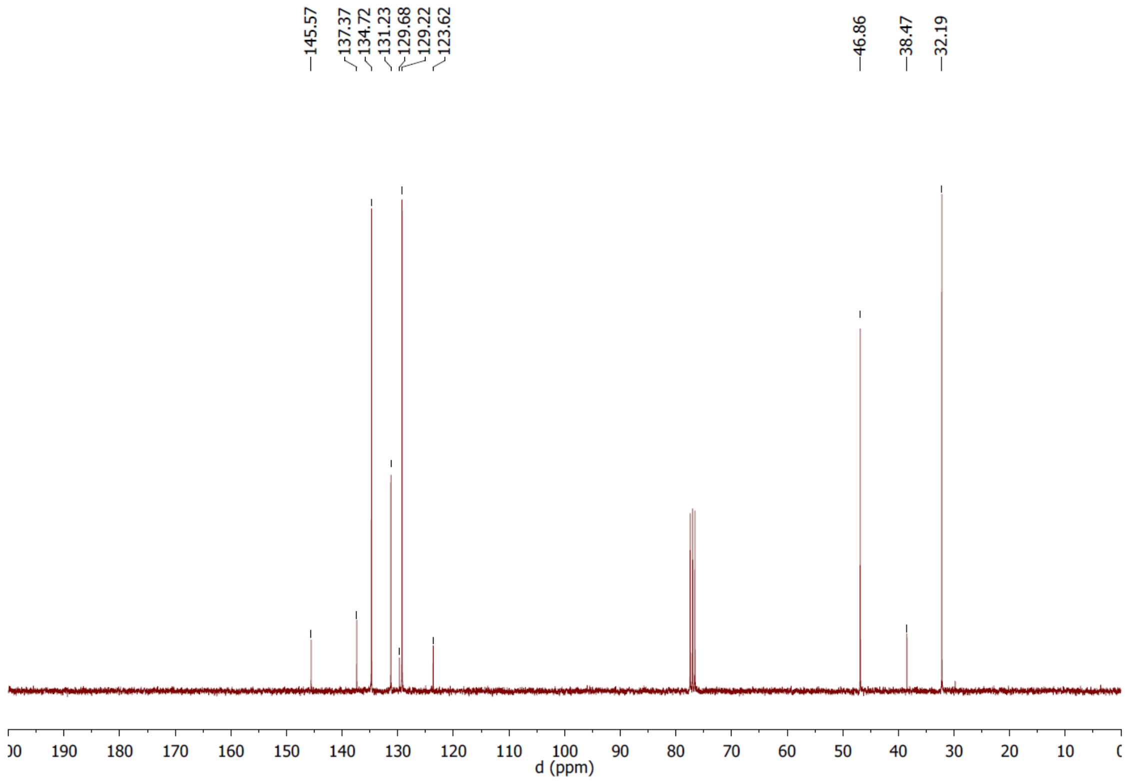

Figure S24. The ^{13}C{^{1}H} NMR spectrum of **12** (75 MHz, $CDCl_3$).

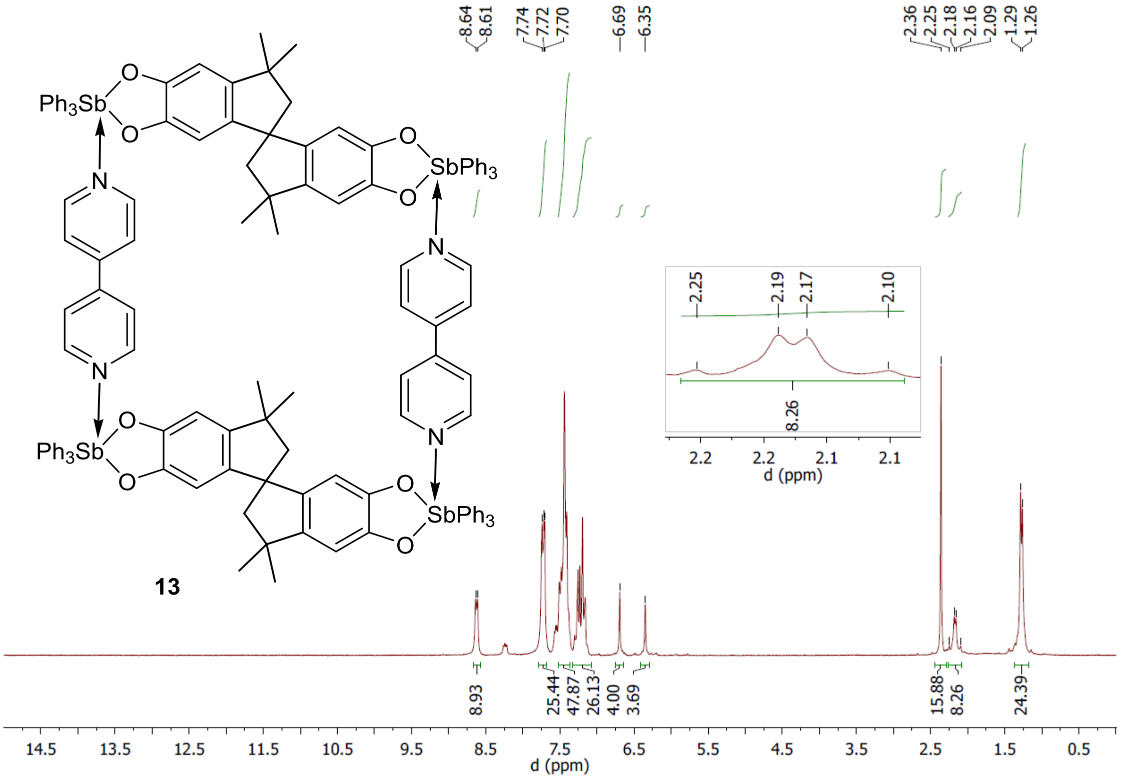

Figure S25. The ^{1}H NMR spectrum of **13** (200 MHz, $CDCl_3$). The peaks of solvated toluene (s. 2.36, m. 7.1-7.3 ppm) are also observed.

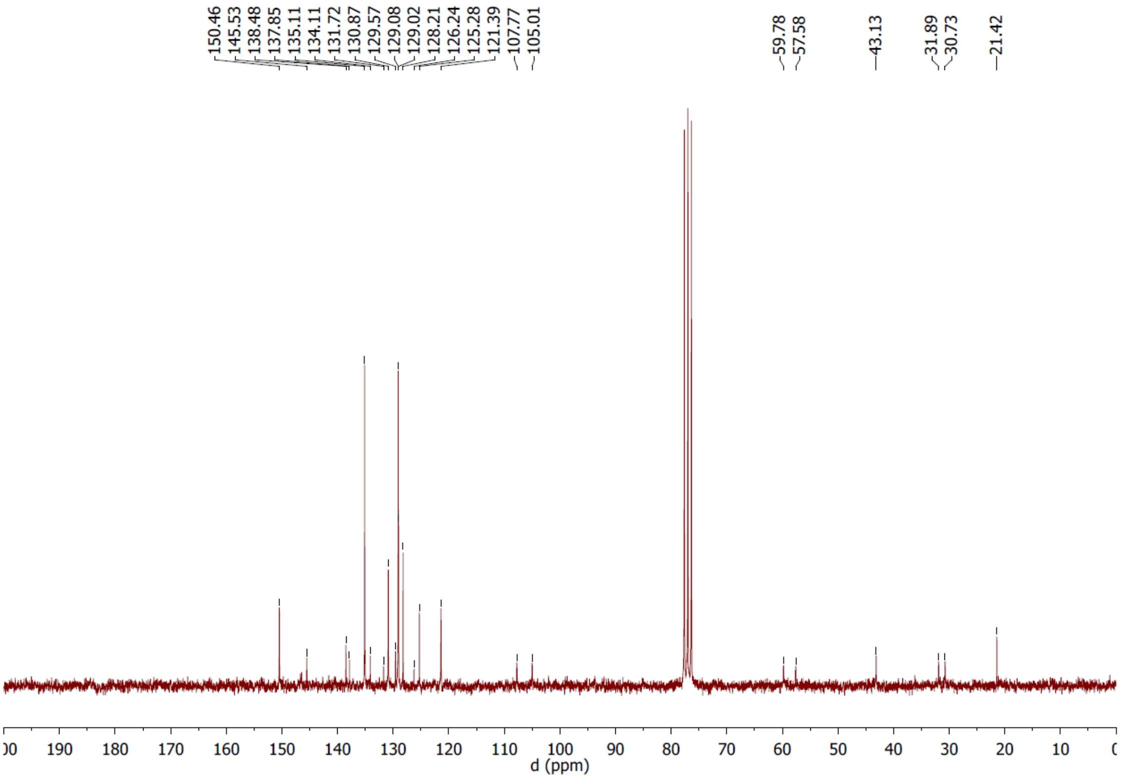

Figure S26. The ^{13}C{^{1}H} NMR spectrum of **13** (50 MHz, $CDCl_3$).

Table S1. The details of X-ray experiment and structure refinement.

Complex	**1**	**3**	**4**·n-hexane	**5**	**8**·toluene	**10**·3.5toluene	**12**·0.25toluene	**13**·6toluene
Empirical formula	$C_{68}H_{74}O_4N_2Sb_2$	$C_{74}H_{78}O_4N_2Sb_2$	$C_{76}H_{96}O_4N_2Sb_2$	$C_{74}H_{78}O_4N_2Sb_2$	$C_{85}H_{94}N_2O_8Sb_2$	$C_{102.5}H_{122}O_4N_6Sb_2$	$C_{39.75}H_{49}Cl_2N_2O_3Sb$	$C_{176}H_{164}N_4O_8Sb_4$
Formula weight	1226.79	1302.88	1345.05	1302.88	1515.12	1745.55	795.46	2950.10
T / K	100(2)	100(2)	100(2)	100(2)	100(2)	100(2)	298(2)	100(2)
Crystal system	Triclinic	Monoclinic	Tetragonal	Triclinic	Triclinic	Triclinic	Monoclinic	Monoclinic
Space group	P-1	P-2(1)/c	P 4(1)/n	P-1	P1	P-1	P-2(1)/c	P-2(1)/c
a/Å	10.1680(4)	14.68919(11)	19.98679(12)	8.39000(10)	12.9130(9)	10.0621(3)	15.7762(6)	14.0936(14)
b/Å	10.2375(4)	13.65117(12)	19.98679(12)	12.5531(2)	16.1438(11)	14.1997(5)	12.8972(4)	27.747(3)
c/Å	14.9255(7)	16.47604(12)	16.97343(16)	15.7830(2)	19.7361(14)	15.5987(5)	19.7694(6)	18.1971(18)
α/°	108.4060(10)	90(5)	90(1)	93.9960(10)	69.4450(10)	74.2840(10)	90	90
β/°	93.5440(10)	102.0369(7)	90.0(2)	105.3940(10)	72.9740(10)	81.9130(10)	94.729(3)	105.380(2)
γ/°	96.6560(10)	90(5)	90.0(5)	100.4650(10)	89.6000(10)	81.7080(10)	90	90
V/Å^3	1456.25(11)	3231.21(5)	6780.40(9)	1563.74(4)	3662.4(4)	2110.86(12)	4008.8(2)	6861.3(12)
Z	1	2	4	1	2	1	4	2
ρ/g·cm^{-3}	1.399	1.339	1.318	1.384	1.374	1.373	1.318	1.428
μ, mm^{-1}	0.978	0.886	0.846	0.915	0.796	0.698	0.858	0.844
F(000)	630	1340	2800	670	1568	913	1642	3032
Q range/°	1.45 – 28.00	2.98 – 30.00	2.88 – 29.99	2.988 – 30.509	2.018 – 28.999	2.057 – 28.763	2.982 – 29.999	2.322 – 25.999
Reflections collected	14573	62216	138260	32429	38096	34127	29785	57188
Uniq. refl. collected	6964	9364	9761	9452	19123	10897	11658	13262
R_{int}	0.0233	0.0321	0.0783	0.0279	0.0327	0.0217	0.0665	0.0695
GOOF (F^2)	1.058	1.038	1.014	1.189	1.020	0.995	1.032	1.025
R_1/wR_2 ($I > 2\sigma(I)$)	0.0291 / 0.0656	0.0212 / 0.0457	0.0315 / 0.0666	0.0423 / 0.1064	0.0382 / 0.0815	0.0281 / 0.0688	0.0668 / 0.1488	0.0392 / 0.0860
R_1/wR_2 (all data)	0.0371 / 0.0678	0.0302 / 0.0478	0.0595 / 0.0722	0.0443 / 0.1072	0.0612 / 0.0872	0.0327 / 0.0710	0.1424 / 0.1754	0.0551 / 0.0904
Largest diff. peak and hole/ e·Å^{-3}	1.532 / -0.479	0.447 / -0.322	0.742 / -0.432	3.687 / -2.079	1.773 / -0.563	2.189 / -1.184	1.650 / -0.796	1.054 / -0.882

Table S2. The selected bond lengths in complexes in the accordance with the bonds scheme.

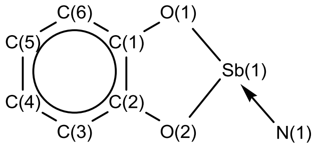	**1**	**3**	**4**	**5**	**8***	**10**	**12**	**13***
Sb(1)-O(1)	2.0194(12)	2.0359(7)	2.0360(10)	2.041(2)	2.0316(17)/ 2.0429(17)	2.0417(12)	2.049(3)	2.020(2)/ 2.009(2)
Sb(1)-O(2)	2.0326(12)	2.0388(7)	2.0405(10)	2.039(2)	2.0291(17)/ 2.0415(16)	2.0124(12)	2.046(3)	2.038(2)/ 2.026(2)
Sb(1)-N(1)	2.773(5)	2.5808(8)	2.645(9)	2.604(3)	2.494(2)/ 2.520(2)	2.727(2)	-	2.439(3)/ 2.463(3)
O(1)-C(1)	1.353(2)	1.3628(12)	1.3672(17)	1.360(4)	1.368(3)/ 1.357(3)	1.360(2)	1.329(6)	1.341(4)/ 1.347(4)
O(2)-C(2)	1.361(2)	1.3607(12)	1.3618(17)	1.364(4)	1.364(3)/ 1.363(3)	1.363(2)	1.327(5)	1.347(4)/ 1.341(4)
C(1)-C(2)	1.409(2)	1.4088(14)	1.415(2)	1.409(4)	1.416(3)/ 1.412(3)	1.427(2)	1.440(6)	1.390(5)/ 1.392(5)
C(2)-C(3)	1.405(2)	1.4086(14)	1.403(2)	1.404(4)	1.399(3)/ 1.404(3)	1.398(2)	1.395(6)	1.375(5)/ 1.365(5)
C(3)-C(4)	1.391(3)	1.3948(15)	1.402(2)	1.407(4)	1.404(3)/ 1.408(4)	1.409(2)	1.429(7)	1.378(5)/ 1.376(5)
C(4)-C(5)	1.389(2)	1.3931(16)	1.386(2)	1.399(4)	1.399(3)/ 1.400(4)	1.395(2)	1.378(8)	1.371(5)/ 1.371(5)
C(5)-C(6)	1.398(2)	1.4004(15)	1.407(2)	1.401(4)	1.407(3)/ 1.394(3)	1.407(3)	1.403(8)	1.376(5)/ 1.378(5)
C(1)-C(6)	1.405(2)	1.3982(15)	1.401(2)	1.389(4)	1.407(3)/ 1.409(3)	1.407(2)	1.424(7)	1.366(5)/ 1.363(4)

* two values correspond to the parameters at/around atoms Sb(1) and Sb(2)

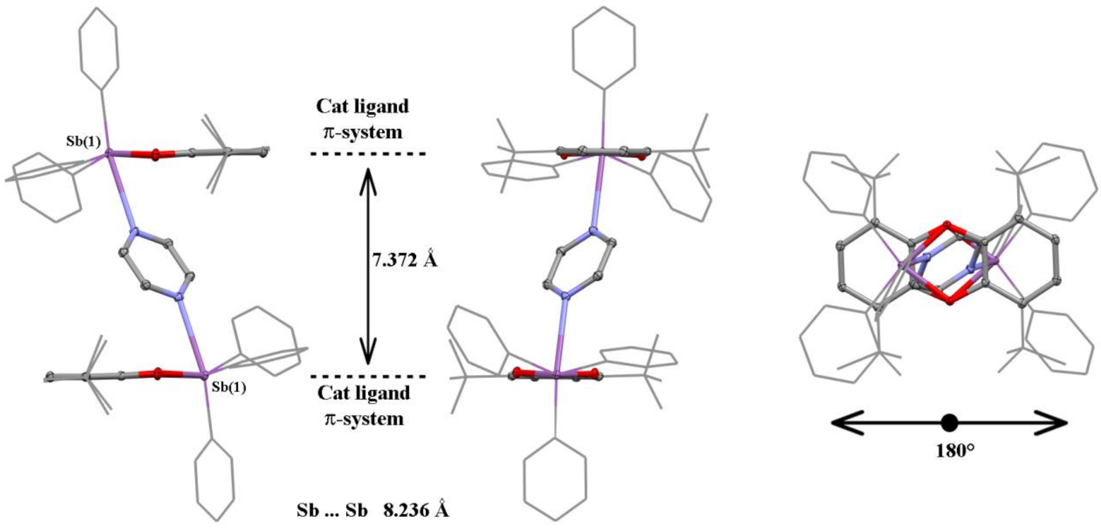

Figure S27. The different views on the molecule of **1** in crystal.

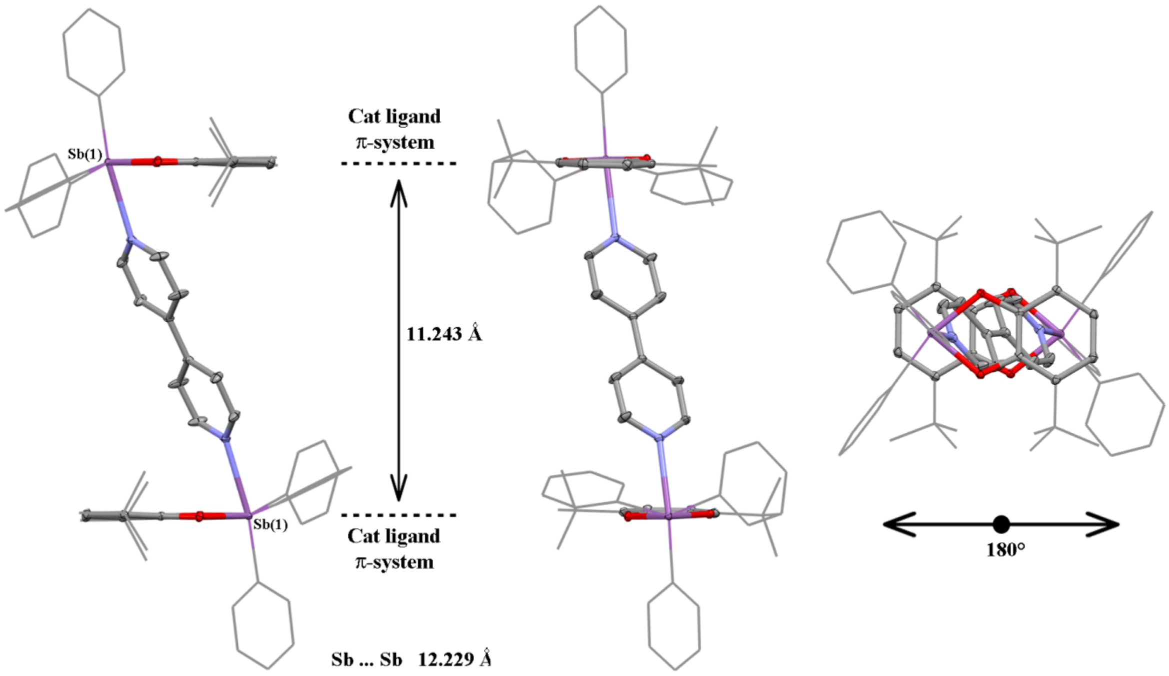

Figure S28. The different views on the molecule of **3** in crystal.

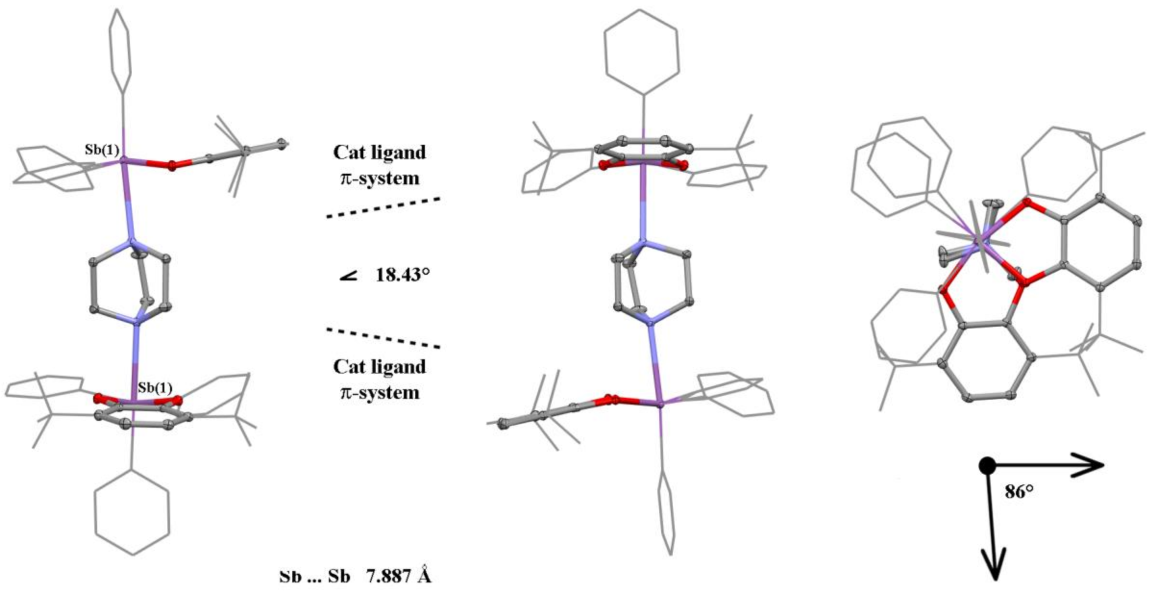

Figure S29. The different views on the molecule of **4** in crystal.

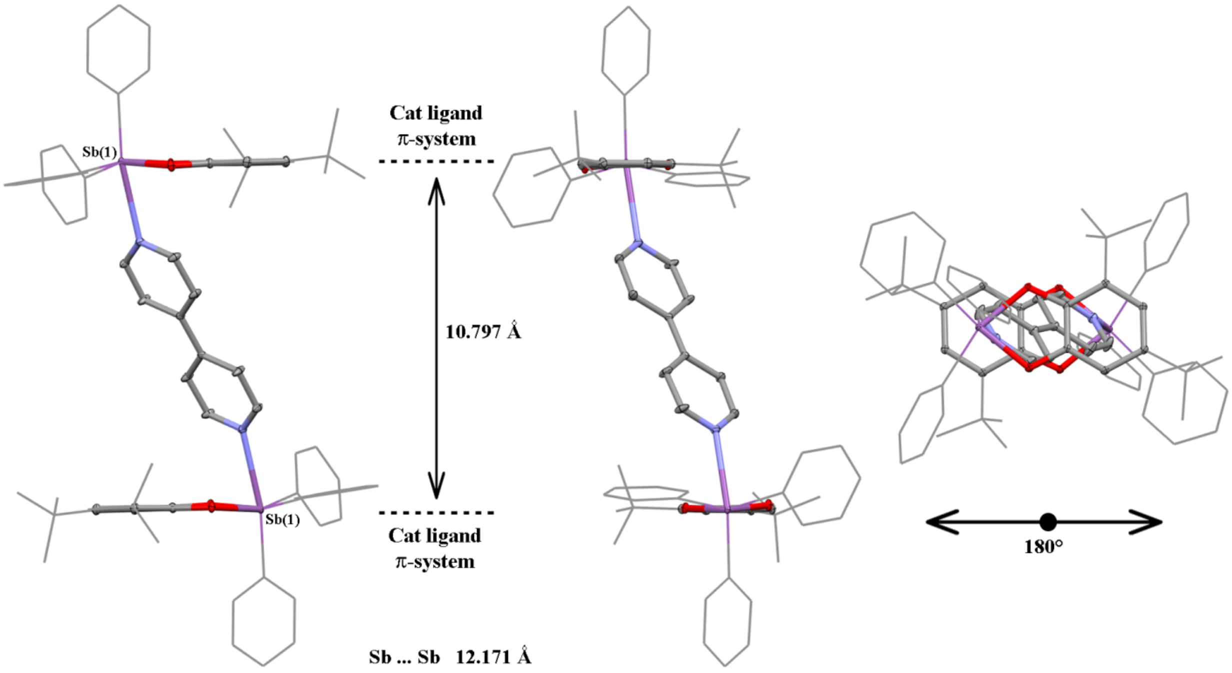

Figure S30. The different views on the molecule of **5** in crystal.

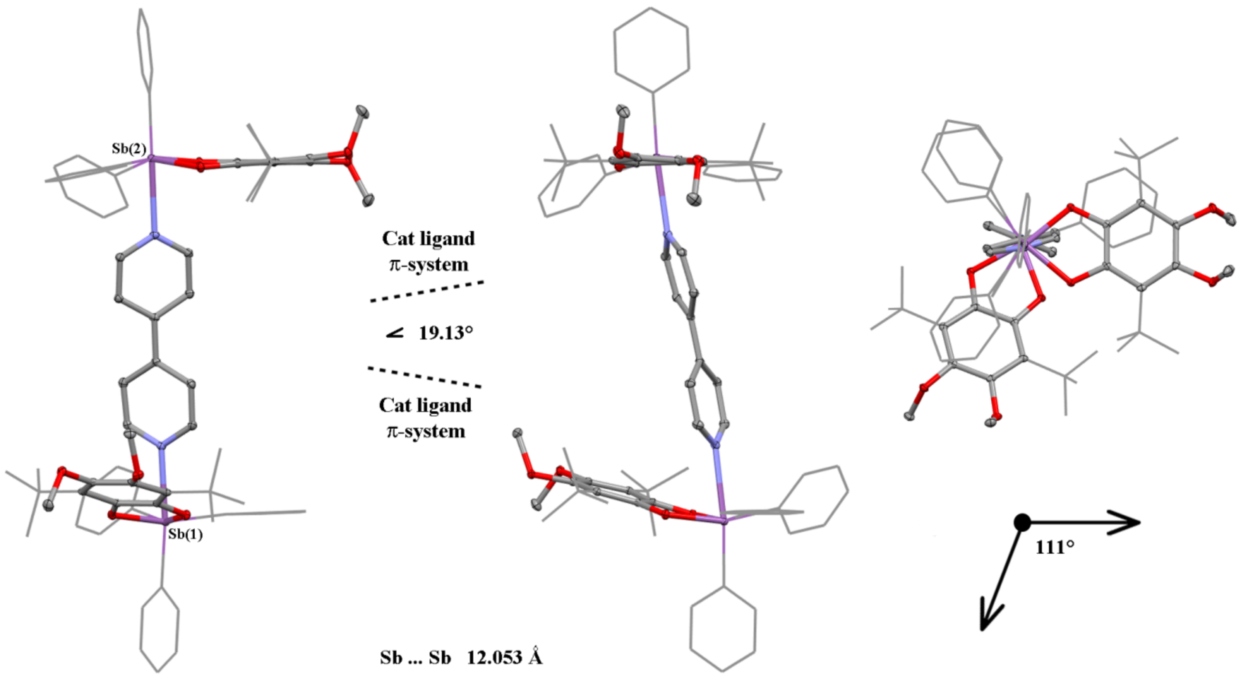

Figure S31. The different views on the molecule of **8** in crystal.

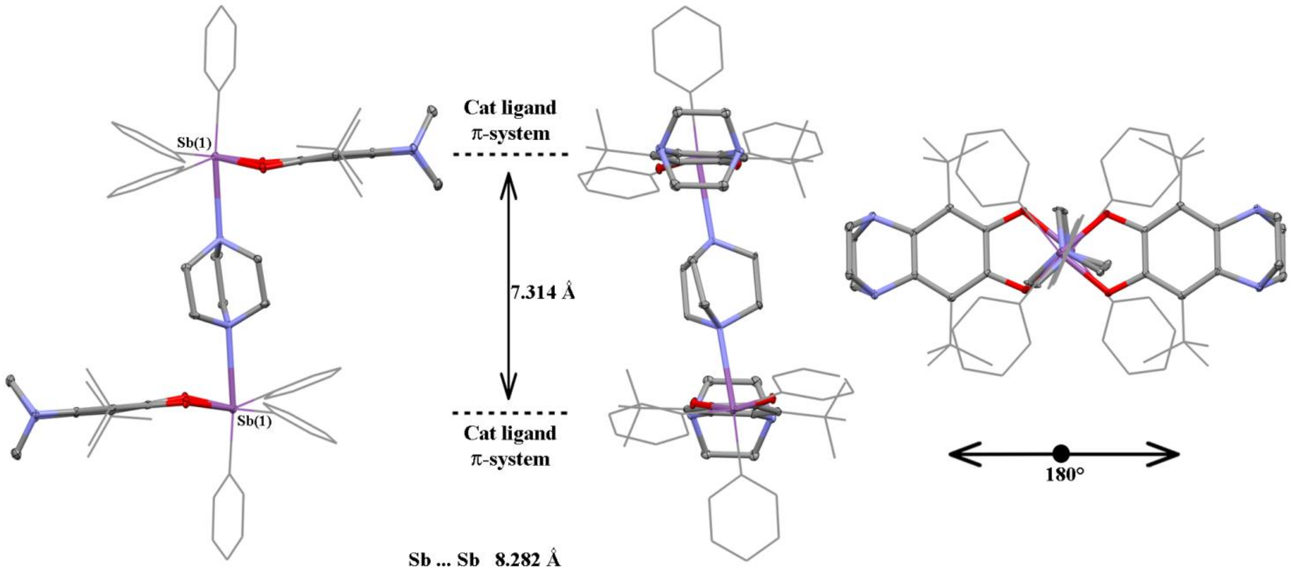

Figure S32. The different views on the molecule of **10** in crystal.

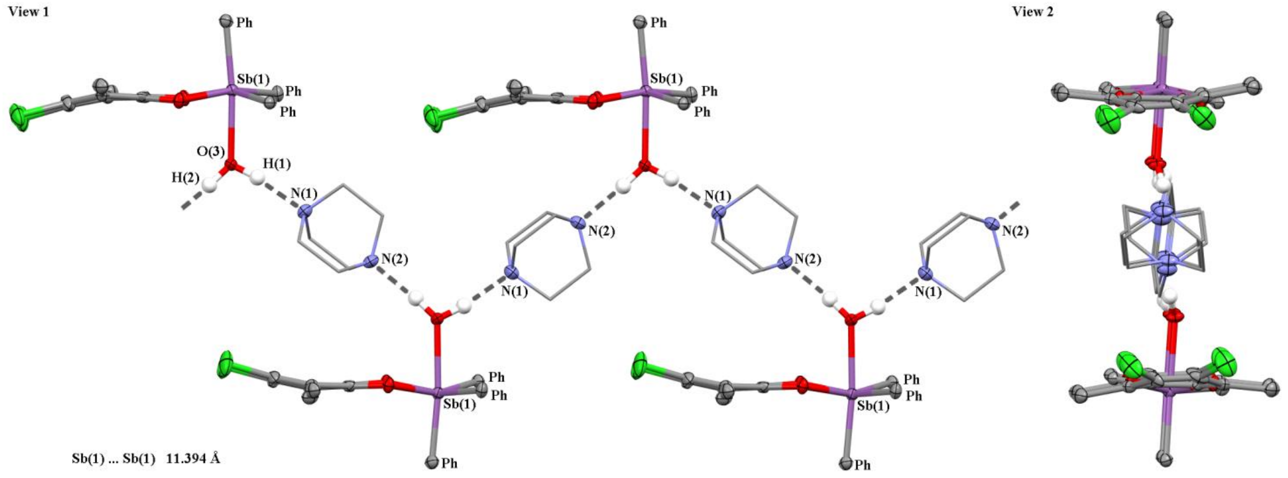

Figure S33. The different views on **12** in crystal.

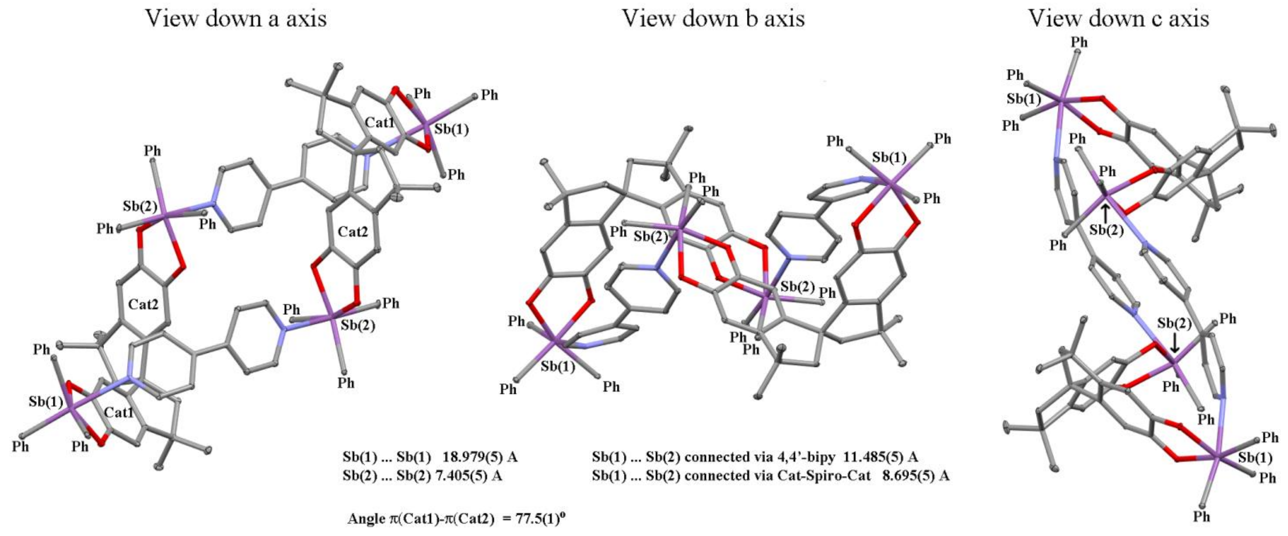

Figure S34. The different views on the molecule of **13** in crystal.

Table S3. The selected structural parameters for bi- and tetranuclear complexes.

Complex / Parameter	**1**	**3**	**4**	**5**	**8***	**10**	**13***
Intramolecular distance Sb...Sb, Å	8.236	12.229	7.887	12.171	12.053	8.282	18.979, Sb(1)...Sb(1), 7.405, Sb(2)...Sb(2), 11.485, Sb(1)...Sb(2) ** 8.695, Sb(1)...Sb(2) ***
Distance Sb...octahedron basal plane, Å	0.349	0.322	0.234	0.283	0.280, 0.318	0.250	0.269, 0.254
Distance Sb...π(Cat) plane, Å	0.085	0.037	0.478	0.131	0.241, 0.273	0.444	0.433, 0.467
Distance between π-system planes of Cat ligands, Å	7.372	11.243	-	10.797	-	7.314	11.608 (Cat1...Cat1), 4.158 (Cat2...Cat2), see Figure S32
Angle between π-system planes of Cat ligands, °	0°	0°	18.43°	0°	19.13°	0°	75.69° (Cat1...Cat2), see Figure S32
Angle between π-system planes of Cat and N-donor ligand, °	54.2°	62.08°	-	57.61°	61.65°, 74.84°	-	37.27° (Cat1...Py) 85.01° (Cat2...Py)
Torsion angle O-C-C-O in chelate cycle, °	0.80°	2.68°	0.34°	2.28°	1.07°, 3.43°	5.59°	0.05° 2.05°
The bent angle of chelate cycle along O...O line, °	5.67°	~0°	17.28°	5.94°	10.39°, 10.06°	18.05°	13.50° 14.82°
The direction of two catecholate fragments	180°	180°	86°	180°	111°	180°	-
Geometry of 4,4'-bipyridine ligand	-	planar	-	planar	nonplanar, angle Py-Py 35.55°	-	nonplanar, angle Py-Py 37.40°

* two values correspond to the parameters at/around atoms Sb(1) and Sb(2);
** for atoms connected via Bipy;
*** for atoms connected via Cat-Spiro-Cat ligand

Multipole refinement of 5_{ED}

The multipole refinement was carried out within the Hansen-Coppens formalism [1] using the MoPro program package [2]. Before the refinement C-H bond distances were normalized to the values obtained in neutron diffraction analyses [3]. The level of the multipole expansion was hexadecapole for terbium atom, octupole for all other non-hydrogen atoms, and one dipole for hydrogen atoms.

The refinement of compound $\mathbf{5_{exp}}$ (θ < 51.43 º) was carried out against F and converged to R = 0.0188, wR = 0.0136, GOF = 0.997 for 32667 merged reflections with $I > 0\sigma(I)$. The ratio of the number of reflections to the number of refined parameters was more than 10 for $\mathbf{5_{ED}}$. All bonded pairs of atoms satisfy the Hirshfeld rigid-bond criteria [4]. Analysis of topology of experimental $\rho(\mathbf{r})$ function was carried out using the WinXPRO program package [5]. The residual electron density is equal 1.11/-1.11 e Å^{-3} for $\mathbf{5_{ED}}$.

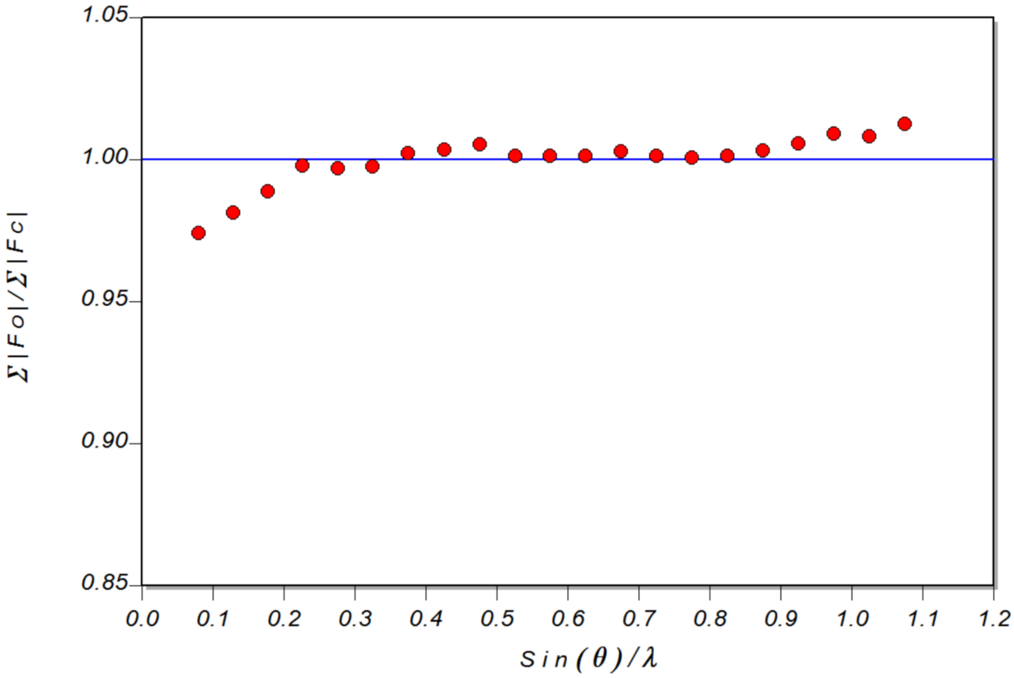

Figure S35. Fo/Fc vs resolution.

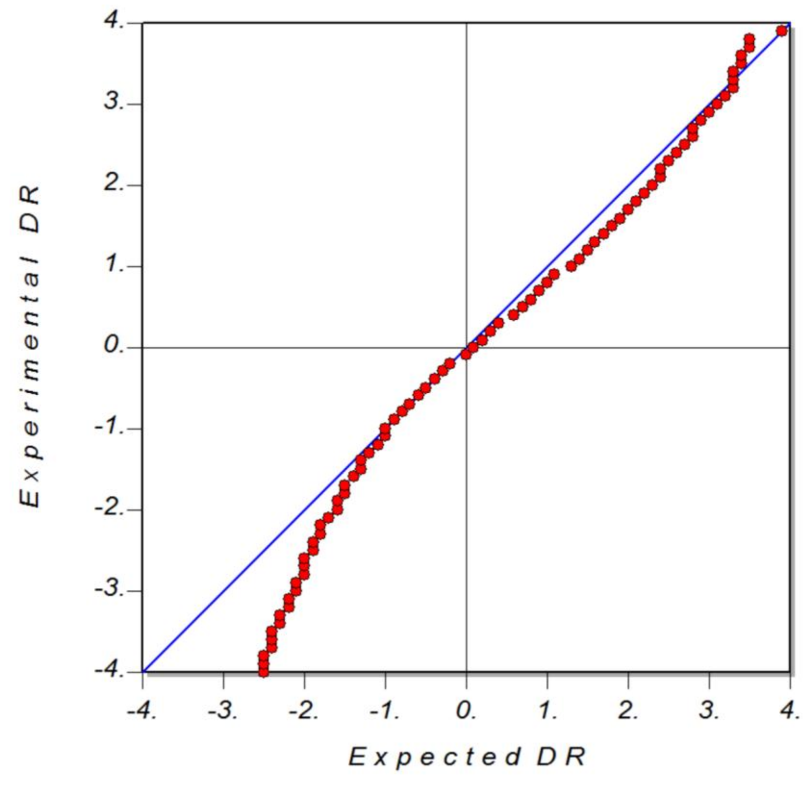

Figure S36. Normal probability.

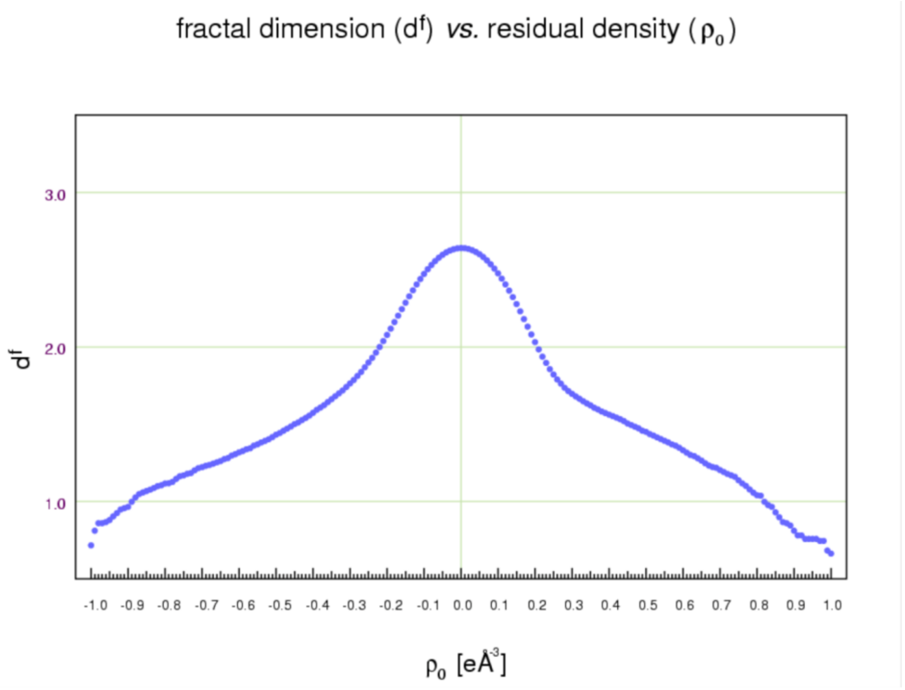

Figure S37. Fractal dimension vs residual density.

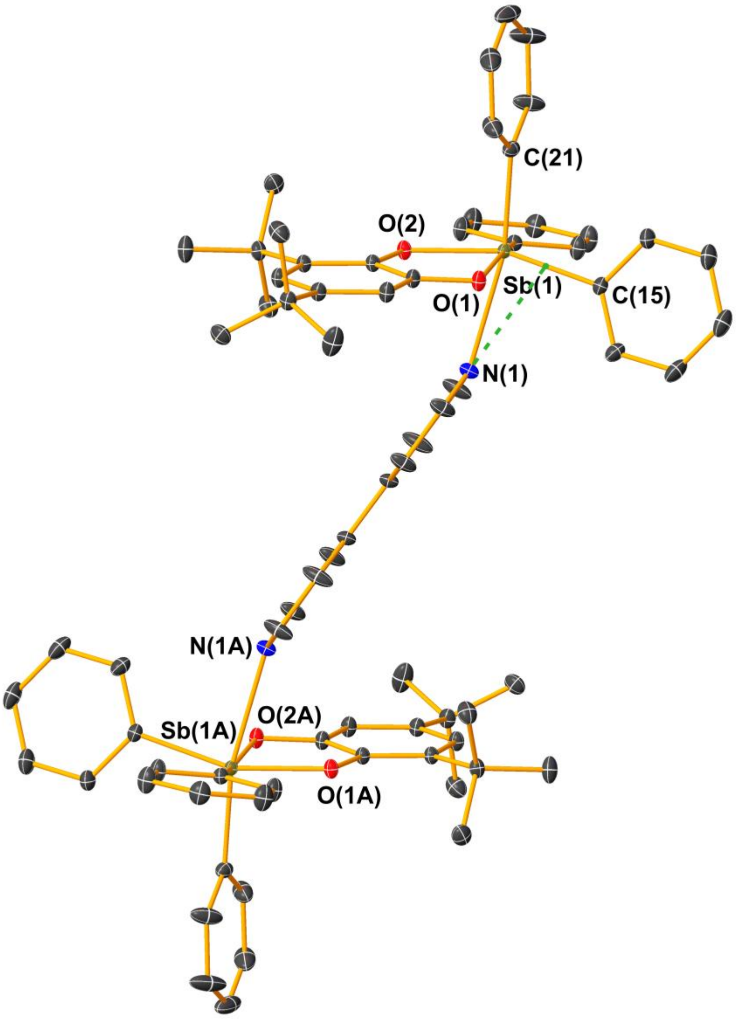

Figure S38. The direction of the lone electron pair of the nitrogen atom in Bipy in **5**$_{\mathbf{ED}}$.

References

1. Hansen, N. K.; Coppens, P. *Acta Crystallogr., Sect. A: Found. Adv.*, **1978**, *34*, 909.
2. Jelsch, C.; Guillot, B.; Lagoutte, A.; Lecomte, C. *J. Appl. Crystallogr.*, **2005**, *38*, 38.
3. Allen, F.H.; Kennard, O.; Watson, D.G.; Brammer, L.; Orpen, A.G.; Taylor, R. *J. Chem. Soc., Perkin Trans.* **1987**, *2*, S1.
4. Hirshfeld, F.L. *Acta Crystallogr., Sect. A: Found. Adv.*, **1976**, *32*, 239.
5. Stash, A.; Tsirelson, V. *J. Appl. Crystallogr.*, **2002**, *35*, 371.
